# Spatio-temporal patterns of juvenile common ravens integrating into a free-flying non-breeder flock

**DOI:** 10.1016/j.isci.2025.114412

**Published:** 2025-12-11

**Authors:** Awani Bapat, Varalika Jain, Christian R. Blum, Palmyre H. Boucherie, Petra Sumasgutner, Thomas Bugnyar

**Affiliations:** 1 Department of Behavioral and Cognitive Biology, University of Vienna, Vienna, Austria; 2 Konrad Lorenz Research Center for Behavior and Cognition, core facility of the University of Vienna, Grünau im Almtal, Austria

## Abstract

For animals living in structured groups, social integration matters: numerous studies reveal fitness advantages for well-integrated over poorly integrated individuals. Surprisingly, little is known about individuals’ spatio-temporal behaviors preceding naturally occurring social integration events. We here applied a biologging-based approach to study juvenile ravens during dispersal, when they approach and join non-breeder groups. We computed three GPS-based movement metrics as proxies for their attraction to locations used by the local non-breeder flock and their attraction to other conspecifics. We found differences in the spatial usage patterns of juveniles compared to older birds, and an influence of the rearing background (wild or captive parents). We also found that familiarity among individuals, i.e., whether they were from the same release groups or not, predicted shared space use. Our results indicate that spatial patterns derived from movement analyses are a promising step toward understanding how individuals spatially orient themselves preceding social integration.

## Graphical abstract

**Graphic abstract:**
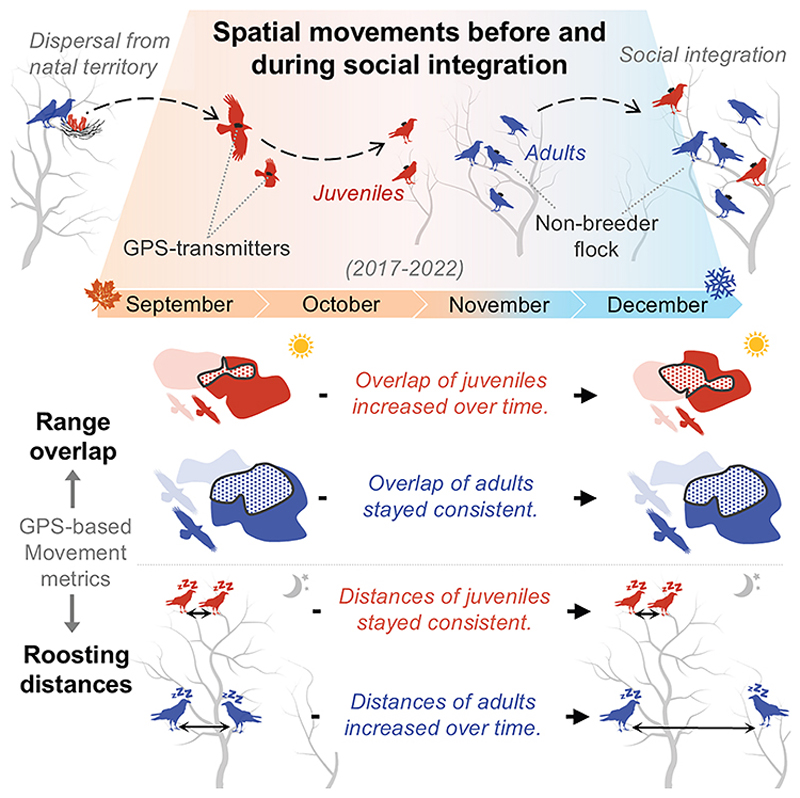

## Introduction

Individuals in socially structured groups vary in the characteristics, numbers, and patterns of relationships that they have with others,^1^ which reflects their degree of social integration in the group.^2^ Being socially integrated has been shown to confer various fitness benefits to individuals, such as survivability^3–5^ and reproductive success.^2,6–12^ However, the costs of maintaining social relationships may also have negative consequences for an individual’s fitness (e.g., lower reproductive success^13,14^ and increased mortality risk^15^). Thus, individuals can be expected to optimize their degree of social integration to maximize their fitness. Furthermore, social environments may vary over time,^16^ with social integration events occurring at key life stages of the individual (e.g., the initial integration after natal or sexual dispersal), or due to group fission or fusion events. To understand how individuals adjust and optimize their social integration following such events, it is important to take a closer look at the behavioral processes involved, i.e., how individuals join groups, actively participate in group social dynamics, and form or strengthen social relationships.

One way to study the process of social integration is to compare social interaction patterns before and after the occurrence of changes in the group’s social composition. Most studies have done this in captive or semi-captive settings (e.g., transfers between zoos, conservational translocations, wildlife reintroductions, and rehabilitations) by introducing new individuals to a group, or by allowing two groups to merge.^17–24^ There are hardly any studies on the development of social relationships during naturally occurring social integration events, such as when juvenile vervet monkeys (*Chlorocebus* pygerythrus)^25^ or yellow-bellied marmots (*Marmota flaviventris*)^26,27^ integrate into their natal groups. Nevertheless, these studies highlight how social interactions during integration may be facilitated by familiarity with group members,^19–23^ kinship,^24–27^ or social play.^28–30^ However, these strategies may not be suitable for species where young, naive individuals have to socially integrate into new groups following dispersal from their natal territories. Prior to interacting socially, they would have to adjust their spatio-temporal behavior to increase the likelihood of encountering others by aligning with the group’s activity patterns and adapting to the local environmental and social contexts.

For studies in the wild, looking into proximate mechanisms, such as movement patterns preceding the formation of social relationships can provide a way to understand social integration without depending on controlled settings or hard-to-collect social interaction data. For example, prior to dispersal, young male vervet monkeys visit home ranges of other groups and affiliate with individuals from neighboring groups during intergroup interactions,^31^ which may allow them to familiarize themselves with the local environmental and social contexts.^32^ Gould^33^ reported movement patterns of two ring-tailed lemurs (*Lemur catta*) as they emigrated from one group and joined a new one, gradually moving from group periphery to the center. Recently, Wild et al.^34^ compared the spatial overlap and social networks during the dispersal and social development of young great tits (*Parus major*), showing that early-life associations and shared space use predicted later social network positions.

Recent advances in GPS-based animal tracking technology have broadened the methodological scope for investigating social behavior in species that range over large distances or in areas that may be inaccessible for human observers to follow.^35–39^ For instance, GPS-based movement data have enabled studies correlating space use (home ranges, sleeping sites, or dens) with socioecological factors (such as group fission-fusion patterns).^40–44^ Other studies have been able to determine association patterns among individuals.^45–48^ However, spatial movement data have rarely been explored in the context of social integration (e.g., Frère et al.^49^ found that home range was positively correlated with female association patterns in bottlenose dolphins, *Tursiops aduncus*).

The recent use of this technology in free-flying common ravens (*Corvus corax*) has elicited key insights into their fissionfusion dynamics^50^ and foraging behavior.^51–54^ Common ravens exhibit a social system wherein pair-bonded adults defend large territories year-round^55^ while juveniles, sub-adults and non-breeding adults form dynamic flocks at foraging and roost sites. The size and composition of these flocks vary within a day, over several days, and across seasons.^50,56–58^ Despite their fluid character, non-breeder groups are structured by dominance relationships and social bonds.^57,59^ Newcomers joining these flocks, such as juveniles leaving their parents’ territory, first face the challenge of finding and approaching these groups, composed of (essentially) unknown individuals and then over-coming the aggression received from the established group members.^59^ This makes them an ideal system for studying the process of social integration.

**Figure 1:**
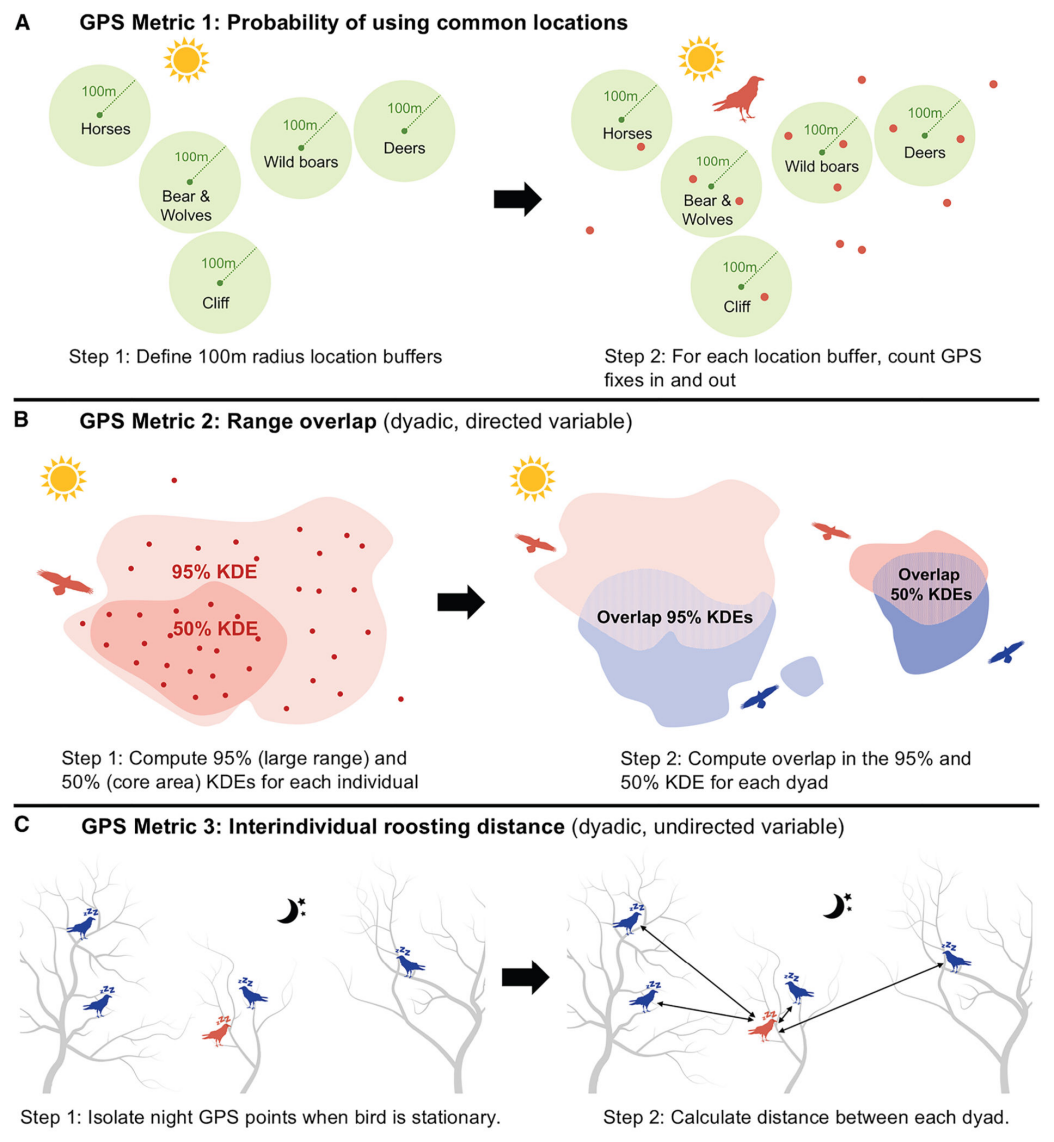
Summary of analysis steps for the computed GPS metrics (A) Probability of using common locations in the wild park (metric 1), computed on a daily scale. (B)Range overlaps in the 95% (large range) and 50% (core area) ranges (metric 2), computed on a biweekly or 14-day periods scale. (C)Interindividual distances during roosting (metric 3), computed on a daily scale.

In this study, we focused on the spatial movement component of social integration that may precede and enable social interactions, and eventually the formation of differentiated social relationships. We sourced movement data from 156 ravens that were GPS-tagged between 2017 and 2022 to quantify the spatio-temporal patterns of juvenile ravens during the early social integration period (from autumn to mid-winter; hereafter, “integration period”) into a local non-breeder group. We computed three GPS-derived metrics (Figure 1) to assess the juvenile ravens’ attraction either to the areas used by local, older ravens, or to other conspecifics. With the first metric, we aimed to investigate whether the juveniles acquire information about the foraging and socializing locations of the local population and adjust their movement patterns to align with them. Specifically, we evaluated the probability of using these key activity areas by computing the daily number of GPS fixes inside and outside five commonly used locations^54,58^ (Metric 1). For the other two metrics, we focused on how ravens positioned themselves in space relative to other group members of the flock. Specifically, we computed the interindividual overlap for two range estimates (95% range, extent of spatial exploration, and 50% range, core range area; Metric 2), and the distance to other individuals during roosting (Metric 3).

Our first question (QI) asked if the spatio-temporal patterns vary among the different age classes of our population, and if the juveniles over time align with the spatial patterns of older birds in the local group. We expected that the spatial usage patterns of juvenile ravens would differ from those of older ravens, and that the patterns of juveniles would change over the integration period to conform to those of the older ravens (reflecting social integration). A special feature of our study population is that it comprises ravens from both wild and captive parents; the latter being raised by their parents in large aviaries and allowed into free flight as juveniles, at approximately the same age as their wild counterparts emigrate from their natal territories. Hence, our second question (QII) asked if individuals from wild or captive parents show variations in the three proxies, given the differences in their rearing environments. Note that for the individuals from captive parents, we have full background information about their upbringing (e.g., family size, parent identity and kinship, and release group when transitioned into free fight). Variations in the early social environments experienced by individuals can result in developmental differences^60^ that may also be reflected in their social integration (e.g., zebra finches, *Taeniopgygia guttata*, from larger broods had higher social network centrality and greater gregariousness during foraging^61^). Further, given that familiarity facilitates social integration,^19–22,24,26,27^ we would expect that familiar individuals (from the same peer groups in captivity) would align with each other’s spatial movements more than with unfamiliar individuals. Thus, our third question (QIII), addressed whether the specific early social conditions experienced by captive young (specifically family size and familiarity status) explained any variations in their spatial usage patterns. While we had no concrete predictions about differences due to wild or captive rearing environments, we expected to see effects of family size in case of the probability of using the common locations (Metric 1), and familiarity status (whether they are siblings, or from same release group or not) in case of range overlap (Metric 2) and interindividual roosting distance (Metric 3). We predicted that birds from large families experiencing enriched early social environments would use the five common locations more than those from small families, and siblings and individuals from same release groups would have a higher overlap in their core ranges; and shorter interindividual roosting distances than unfamiliar dyads.

## Results

**Figure 2:**
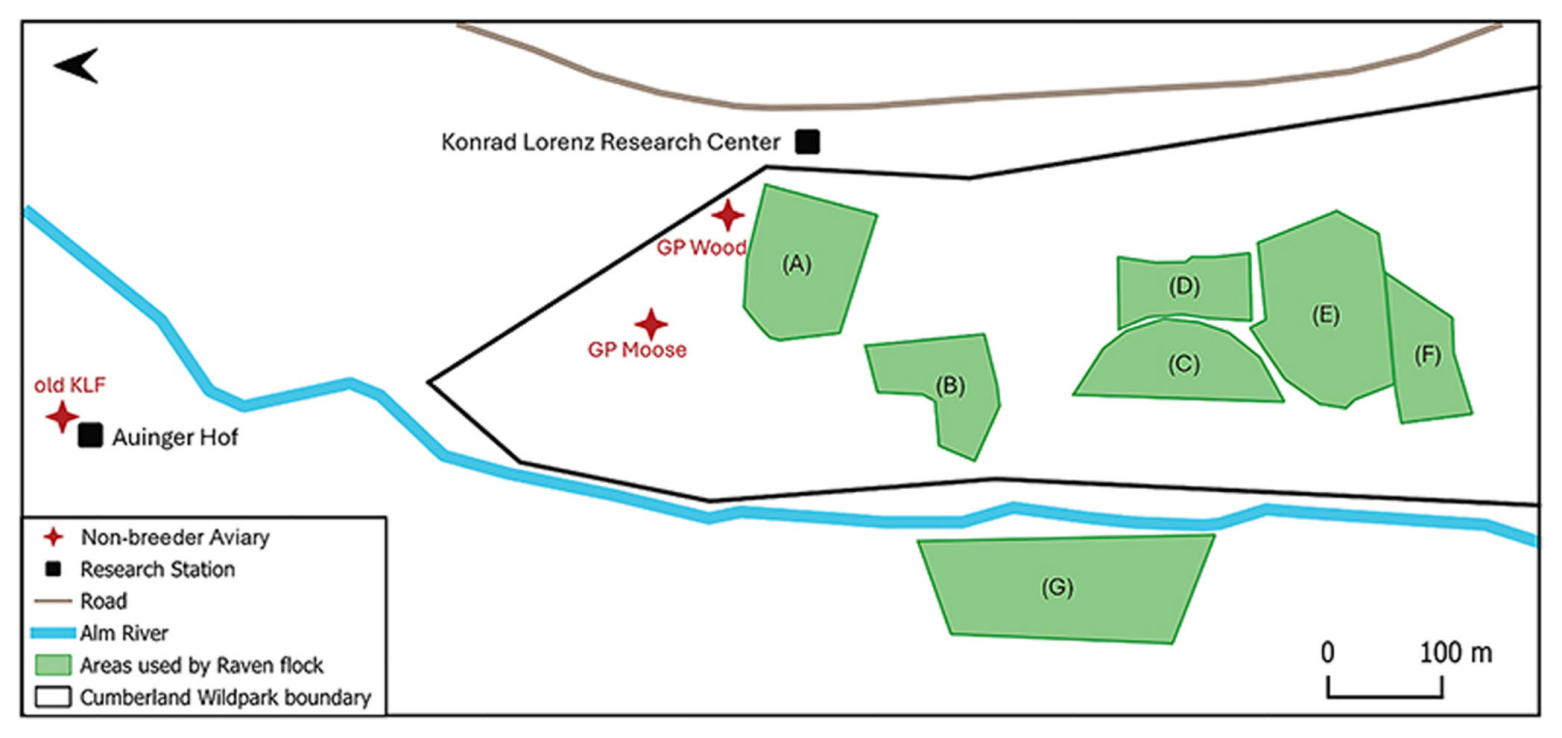
Map of the study area Map shows the Konrad Lorenz Research Center and Auinger Hof (historic research center) adjacent to the Cumberland Wildpark; the non-breeder aviaries from which the juveniles from captive parents were allowed into free flight (old KLF: 2018–2021, GP Wood: 2017–2022, and GP Moose: 2022); and the enclosures of Przewalksi’s horses (A), bears and wolves (B), wild boars (C), chamois (D), fallow deer (E), and roe deer (F), and the cliff (G) which are commonly used by the raven flock.

The three spatial use metrics were derived from GPS-tagged individuals at our study site, the Cumberland Wildpark in the Northern Alps, Grünau im Almtal, Austria (Figure 2). Figure 1 briefly summarizes the three computed metrics; refer to STAR Methods for details. To examine variations across age class (QI) and rearing background (QII), statistical analyses were carried out on the entire dataset. Note that Metric 2 (range overlap) was a directed, dyadic variable and was analyzed in two parts: first, we modeled the probability of an overlap occurring as a binary response, and second, for observations where an overlap occurred, we modeled the magnitude of the observed overlap as the response. To examine how the social conditions (family size, kinship, and release group identity) experienced by individuals raised by captive parents affected the three metrics (QIII), we used a subset including only individuals from captive parents. Tables S1–S6 provide details of the effective sample sizes for each of the analyses.

To analyze variations in spatial use patterns across age classes (QI) and different rearing backgrounds (i.e., origin from wild or captive parents, QII), using our three metrics we fitted four generalized linear mixed models ([GLMMs], Table 1). Each model incorporated the interaction between age class and time (within the integration period, QI). Depending on the response variable, age class was defined at the individual (Metric 1, Model 1a) or dyadic level (Metrics 2 and 3, Models 2a and 2c, and 3a, respectively; hereafter “age class pair”), and time was measured in number of days (Models 1a and 3a) or biweek period (Models 2a and 2c). We also included the interaction between origin — either at the individual (Model 1a) or dyadic level (Models 2a, 2c, and 3a, hereafter “origin pair”) — and the corresponding time unit (as described above, QII). To differentiate between the effects of the test predictors on the overlap in the 50% versus the 95% ranges in Models 2a and 2c, we included overlap type in three-way interactions with age class and time (QI), and origin and time (QII). For Model 1a, we also included the interaction between individual age class and origin. For all the above models, the comparisons between the full and the null models (including only the control fixed predictors and random effects, see STAR Methods: statistical analyses) were statistically significant (GLMM, Model 1a: χ^2^ = 2,968.306, df = 19, *p* < 0.001; Model 2a: χ^2^ = 738.442, df = 74, *p* < 0.001; Model 2c: χ^2^ = 1,220.882, df = 74, *p* < 0.001; Model 3a: χ^2^ = 602.250, df = 22, *p* < 0.001) confirming that the included test predictors meaningfully contributed to explaining the variability in the respective response variables.

### QI: Variations in spatial use patterns across age classes

**Figure 3:**
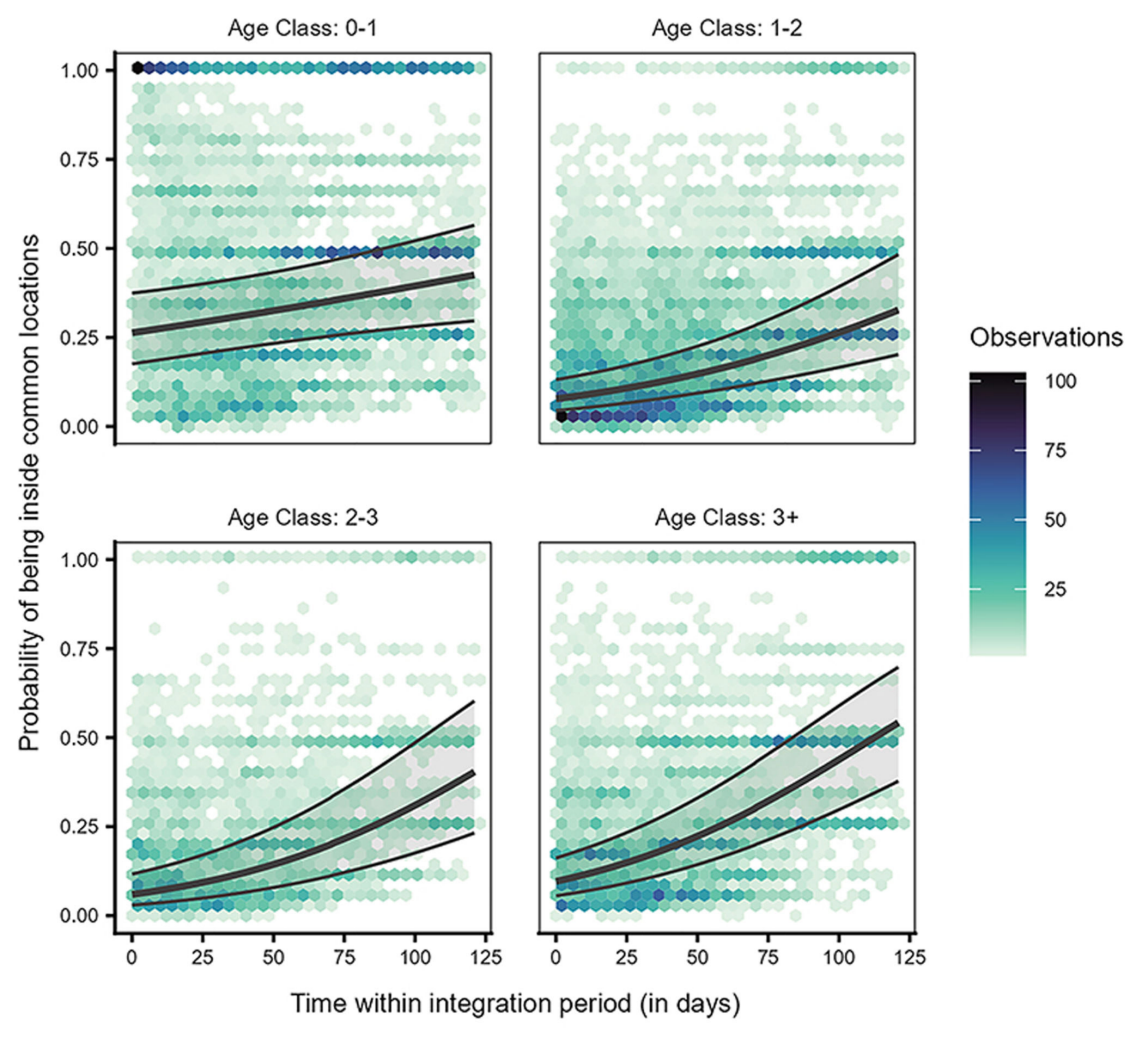
Juvenile ravens differed from older birds in their use of the common locations over time Probability of being present inside any of the five locations commonly used by the local free-flying population for individuals of different age classes (0–1: juveniles, 1–2: sub-adults year 1, 2–3: subadults year 2, 3+: adults) over time within the integration period (QI, Model 1a). The hexagon plot depicts the observed data distribution, with the hexagons’ colors indicating the number of observations in each hexagonal bin. The ribbons indicate the 95% confidence intervals around the estimated adjusted predictions (thick line).

Model 1a revealed a significant interaction between individual age class and time (likelihood ratio test [LRT]: χ^2^ = 22.102, df = 3, *p* < 0.001, Table S7), indicating that individuals of different age classes varied in their temporal use patterns of the locations commonly used by the local population (i.e., the horse, bear and wolf, wild boar, and deer enclosures, and the cliff) over the integration period. For the most part, juveniles (0–1 year) were present in the locations more than the older birds (sub-adults year 1: 1–2 years, sub-adults year 2: 2–3 years, adults: 3+ years); however, the increase in presence within the locations over the integration period was steeper for the older birds than for the juveniles (Figure 3, Table S7).

**Figure 4:**
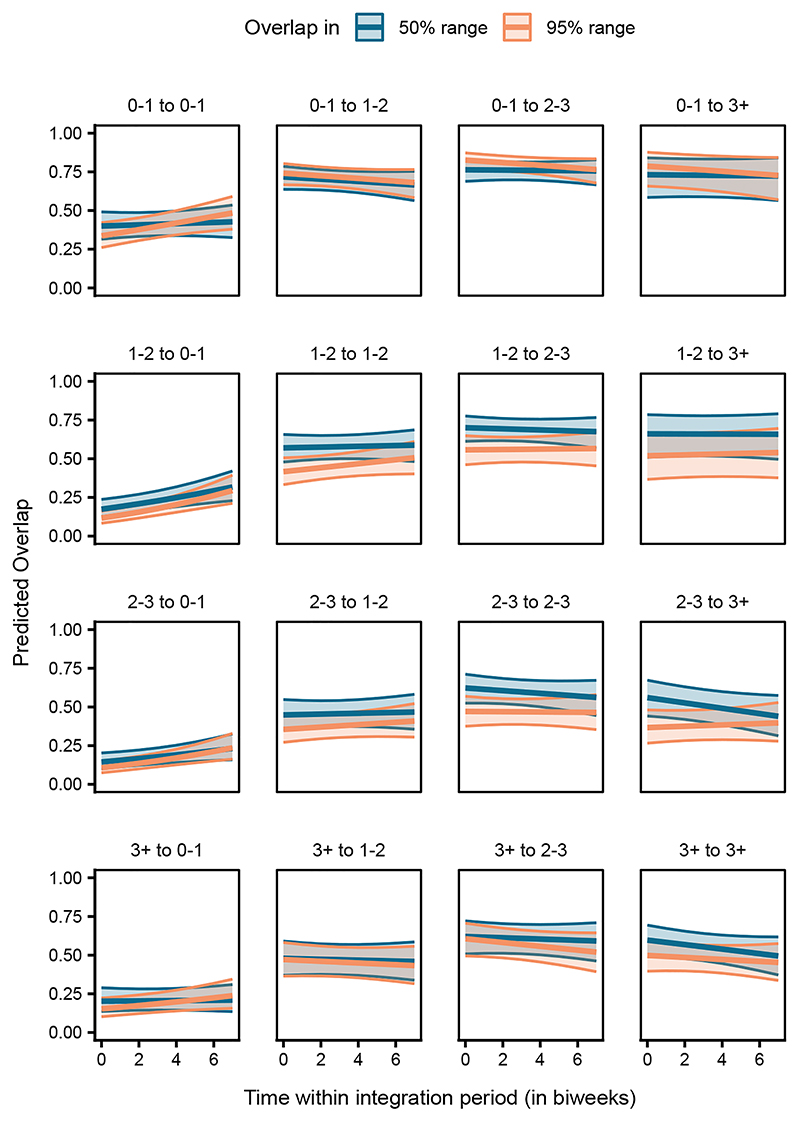
Juveniles showed greater overlap with older ravens, while older ravens overlap less with juveniles Predicted overlap in the 50% (dark blue) and 95% (light orange) ranges for dyads of individuals of different age classes (0–1: juveniles, 1–2: subadults year 1, 2–3: sub-adults year 2, 3+: adults) over time within the integration period (QI, Model 2c). The *y*–axis represents the dyadic, directed response variable. The ribbons indicate the 95% confidence intervals around the estimated adjusted predictions (thick line). See Figure S2 for observed data distribution.

In the case of range overlap, the interaction between age class pair, overlap type, and time significantly explained the probability of overlap occurrence and the magnitude of overlap among the dyads (LRT, Model 2a: χ^2^ = 325.653, df = 15, *p* < 0.001, Table S8; Model 2c: χ^2^ = 96.657, df = 15, *p* < 0.001, Table S9). Overall, for dyads of all age class combinations, the probability of an overlap occurring in the 95% range was consistently very high, whereas the probability of an overlap occurring in the 50% range varied greatly among the different age class pairs (Figure S1). However, given the large confidence intervals (Table S8, Figure S1), these results must be interpreted with caution. Hence, we do not discuss the differences between the age class pairs here (see STAR Methods for explanation). Nevertheless, for cases where an overlap occurred, the magnitude of overlap of juveniles with older birds (sub-adults year 1, subadults year 2, and adults) was higher than that with other juveniles, in both the 50% and 95% ranges (Figure 4). The magnitude of overlap of juveniles with the older birds was also fairly stable over time within the integration period (Figure 4). On the contrary, older birds overlapped with the juveniles much less than they did with other older birds, in both the 50% and 95% ranges (Figure 4). Comparing the same-aged dyads, juveniles partially overlapped in the 50% range consistently, but their overlap in the 95% range increased over time. In contrast, older birds’ dyads remained relatively consistent in both the 50% and 95% ranges (Figure 4).

**Figure 5:**
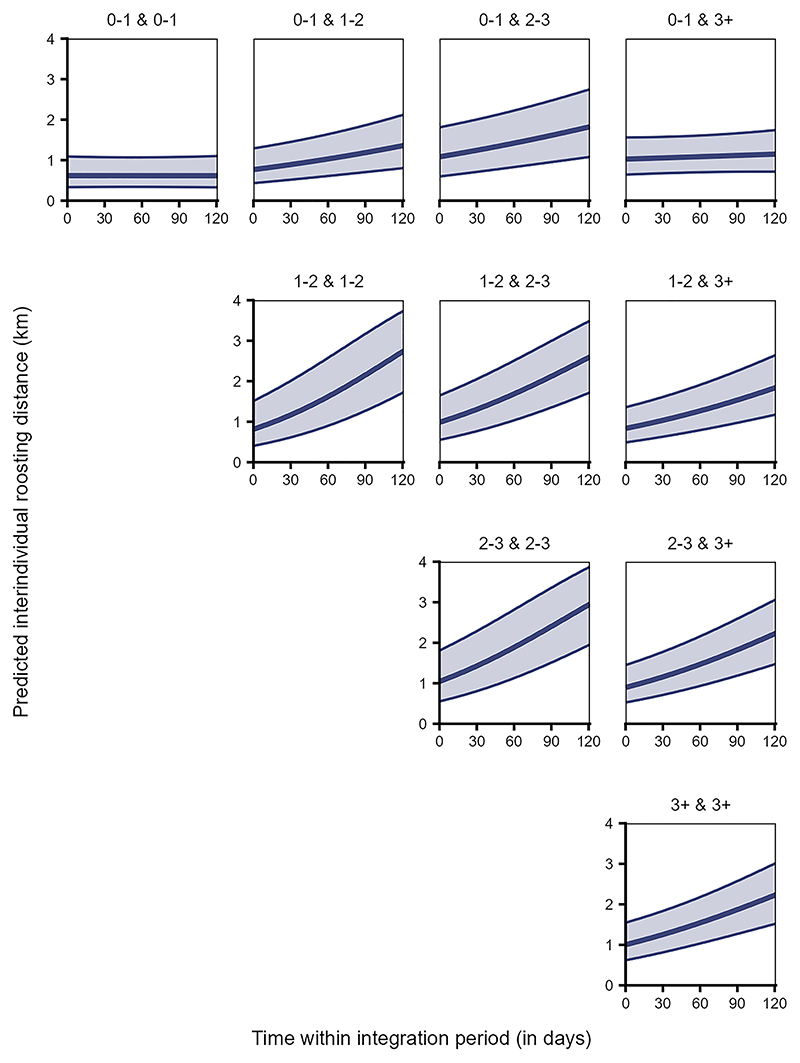
Juveniles roosted closer to other juveniles, while older birds varied in their roosting proximity to others over time Interindividual roosting distances for dyads of individuals of different age classes (0–1: juveniles, 1–2: sub-adults year 1, 2–3: sub-adults year 2, 3+: adults) over time within the integration period (QI, Model 3a). The *y*–axis depicts the back-transformed response variable (dyadic, undirected), which was statistically modeled as a continuous proportion. The topmost row depicts all dyads of which at least one individual is a juvenile. The ribbons indicate the 95% confidence intervals around the estimated adjusted predictions (thick line). See Figure S3 for the observed data distribution.

Further, the interaction between age class pair and time significantly explained the variation in interindividual roosting distances (Model 3a, LRT: χ^2^ = 546.707, df = 9, *p* < 0.001, Table S10). Juveniles roosted closest to other juveniles and then to adults consistently through time, whereas the interindividual roosting distances between juveniles and sub-adults increased over time (Figure 5). A similar, but steeper trend was observed among the different dyads of older birds (Figure 5).

### QII: Variations in spatial use patterns of individuals from wild or captive parents

**Figure 6:**
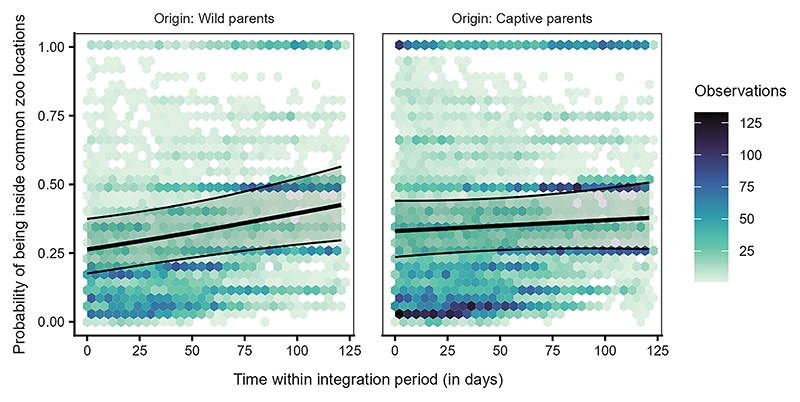
Individuals from wild or captive parents differed in their use of the common zoo locations over time Probability of being present inside any of the five commonly used zoo locations for individuals from wild or captive parents over time within the integration period (QII, Model 1a). The hexagon plot depicts the observed data distribution, with the colors of the hexagons indicating the number of observations in each hexagonal bin. The ribbons indicate the 95% confidence intervals around the estimated adjusted predictions (thick line).

**Figure 7:**
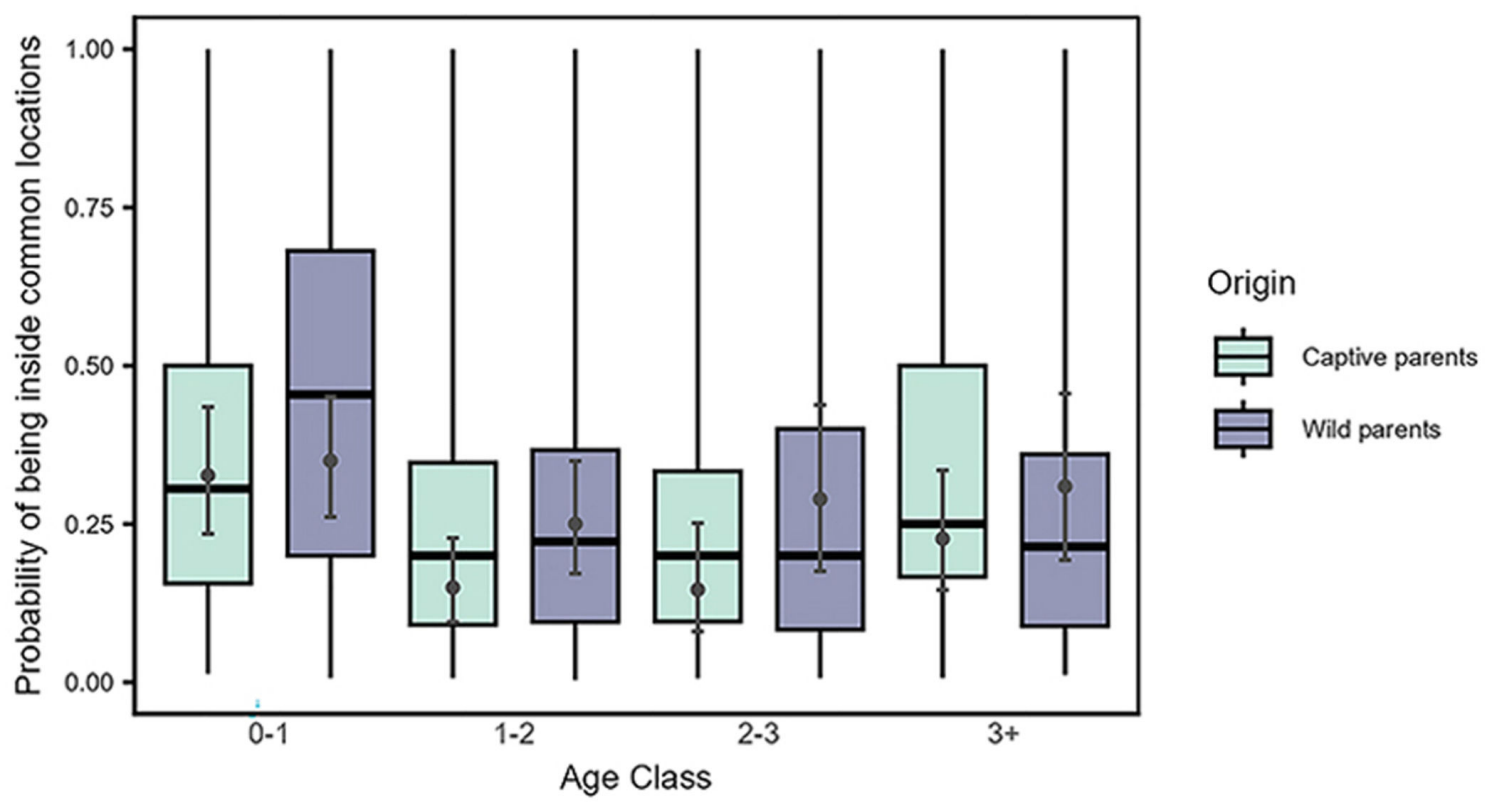
Juveniles, irrespective of their origin, were more likely to use the common zoo locations than the older birds Probability of being present inside any of the five commonly used locations by the local free-flying population across age class (0–1: juveniles, 1–2: sub-adult year 1, 2–3: sub-adults year 2, 3+: adults) and origin (individuals from captive parents in light green and from wild parents in dark violet; QII, Model 1a). Boxplots depict the median, 0.25 and 0.75 quantiles, with the whiskers indicating the range of the observations. Error bars indicate 95% confidence intervals around the estimated marginal means (gray points).

The probability of being present in any of the five commonly used locations, was significantly affected by the interaction between origin and time (LRT: χ^2^ = 3.862, df = 1, *p* = 0.049, Table S7). The ravens from wild parents showed a slightly steeper increase with time in their use of the locations compared to the ravens from the captive parents, who were relatively consistent in their use of the locations (Figure 6, Table S7). Furthermore, the interaction between origin and age class was also significant (LRT: χ^2^ = 22.102, df = 3, *p* < 0.001, Table S7). Irrespective of their origin (wild or captive parents), juveniles were more likely to use the locations than older ravens. However, older individuals from captive parents were more likely to be present in the locations than older birds from wild parents (Figure 7).

**Figure 8:**
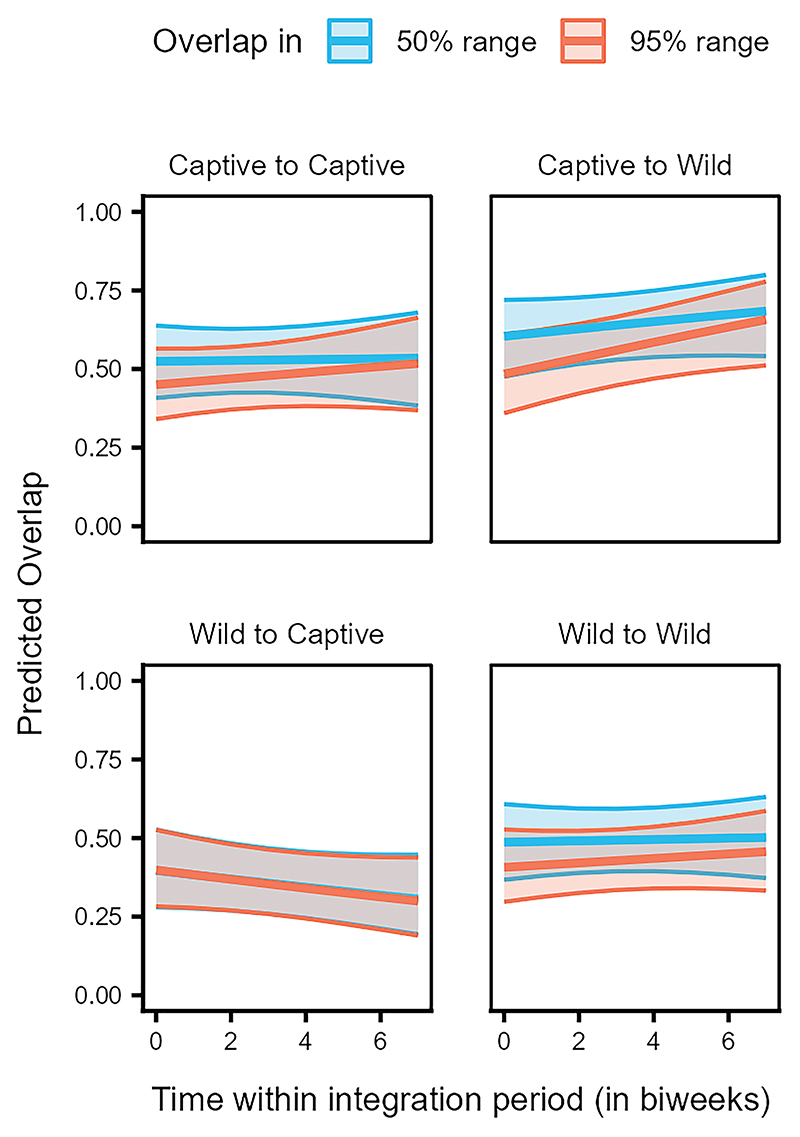
Individuals from captive parents overlap more with those from wild parents, while those from wild parents overlap less with birds from captive parents Predicted overlap in the 50% (dark blue) and 95% (light orange) ranges for dyads of individuals of different origin (from captive or wild parents) over time within the integration period (QII, Model 2c). The *y*–axis represents the dyadic, directed response variable. The ribbons indicate the 95% confidence intervals around the estimated adjusted predictions (thick line). See Figure S5 for observed data distribution.

In the case of range overlap, the interaction of origin pair, overlap type and time, significantly predicted the probability of overlap occurrence and the magnitude of range overlap (LRT, Model 2a: χ^2^ = 8.744, df = 3, *p* = 0.033, Table S8, Figure S4; Model 2c: χ^2^ = 23.466, df = 3, *p* < 0.001, Table S9). The dyads of individuals with the same origins (i.e., captive-captive and wild-wild) showed very similar trends of consistent partial overlaps in the 50% and 95% ranges, with captive-captive dyads overlapping slightly more than wild-wild dyads (Figure 8). In contrast, the overlap of birds from captive parents to those from wild parents in both the 50% and 95% ranges was higher and increased over time, as compared to the overlap of birds from wild parents to those from captive parents, which showed a decreasing trend over time (Figure 8).

**Figure 9:**
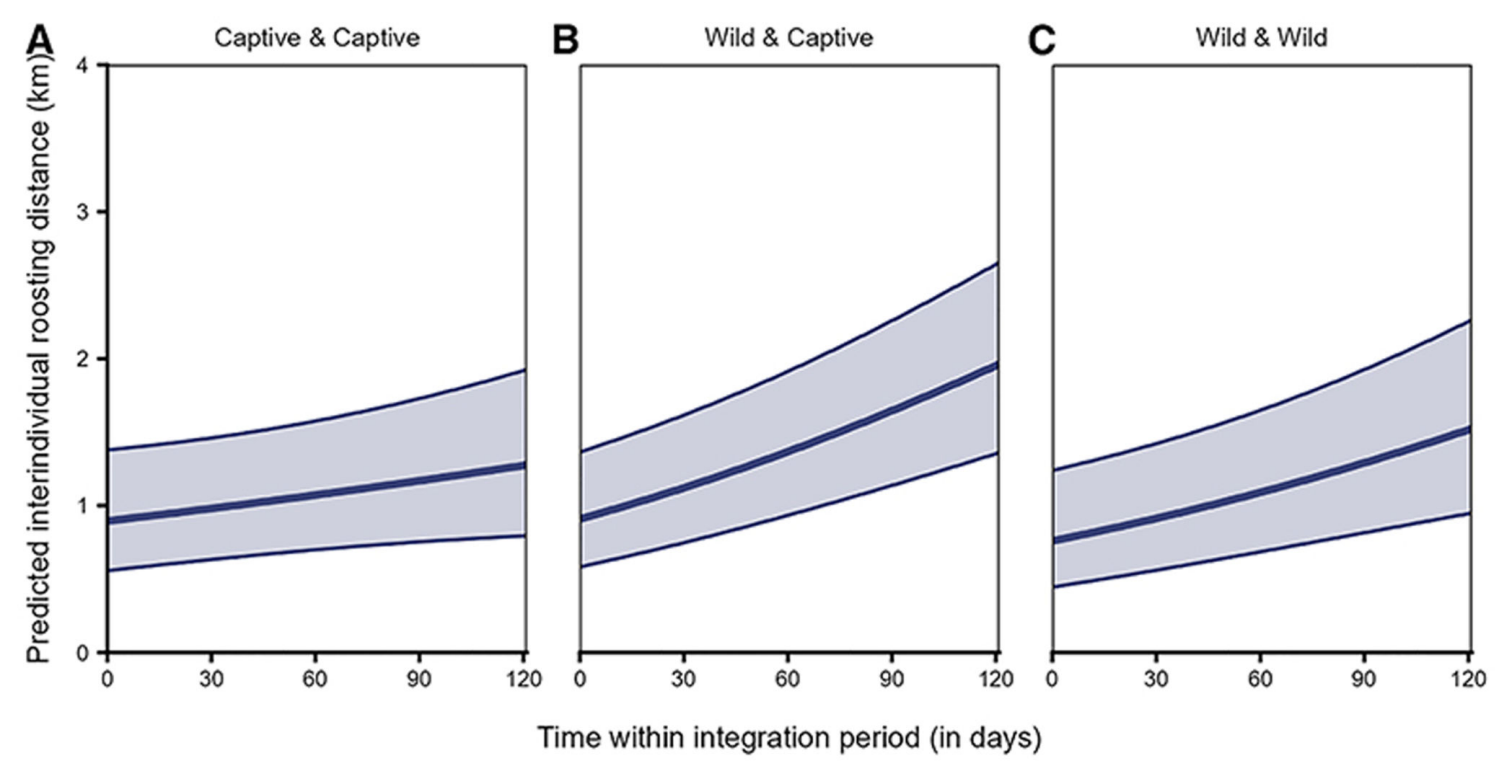
Interindividual roosting distances among mixed dyads and wild dyads increased more steeply over time as compared to the captive dyads Interindividual roosting distances during roosting for dyads of different origin (A) both individuals from captive parents, (B) individuals from captive parents and wild parents, and (C) both individuals from wild parents over time within the integration period (QII, Model 3a). The *y*–axis depicts the back-transformed response variable (dyadic, undirected), which was statistically modeled as a continuous proportion. The ribbons indicate the 95% confidence intervals around the estimated adjusted predictions (thick line). See Figure S6 for the observed data distribution.

The interindividual roosting distances were significantly affected by the interaction of origin pair and time (LRT: χ^2^ = 25.794, df = 2, *p* < 0.001, Table S10). While dyads of all origin combinations initially roosted at similar distances to each other, the increase in interindividual distances over time was steepest for the mixed dyads (i.e., wild-captive dyads), followed by the wild dyads (i.e., both individuals with wild parents), as compared to the captive dyads (i.e., both individuals with captive parents, Figure 9).

### QIII: Effect of early life social factors on the spatial use patterns of ravens from captive parents

To examine how family size experienced by captive-raised individuals affected the three metrics (QIII), we fitted four additional GLMMs (Table 1), in each, including family size, defined at the individual (Metric 1, Model 1b) or dyadic level (Metrics 2 and 3, Models 2b and 2d, and 3b, respectively, hereafter “family size pair”) as the test predictor. For Models 2b, 2d, and 3b, we also included test predictors for familiarity status, i.e., whether the dyads were siblings or not (“sibling status”) and whether they were from the same or different release groups (“release group type”), and their respective interactions with time (QIII). In case of Models 2b and 2d, we included a two-way interaction between overlap type and family size pair, and three-way interactions of sibling status or release group type with time and overlap type, to differentiate between the effects of the test predictors on the overlap in the 50% versus the 95% ranges. The full-null model comparison revealed no significant effect of family size, on the probability to use common locations (GLMM, Model 1b: χ^2^ = 0.072, df = 1, *p* = 0.789). However, in case of the other two metrics, the full-null comparison was significant (GLMM, Model 2d: χ^2^ = 45.970, df = 14, *p* < 0.001; Model 3b: χ^2^ = 93.573, df = 6, *p* < 0.001), suggesting that the early social conditions explained the variance in the range overlap and interindividual roosting distances. As we encountered diagnostic issues with Model 2b, the results were not interpreted and are not shown here (see STAR Methods for details).

**Figure 10:**
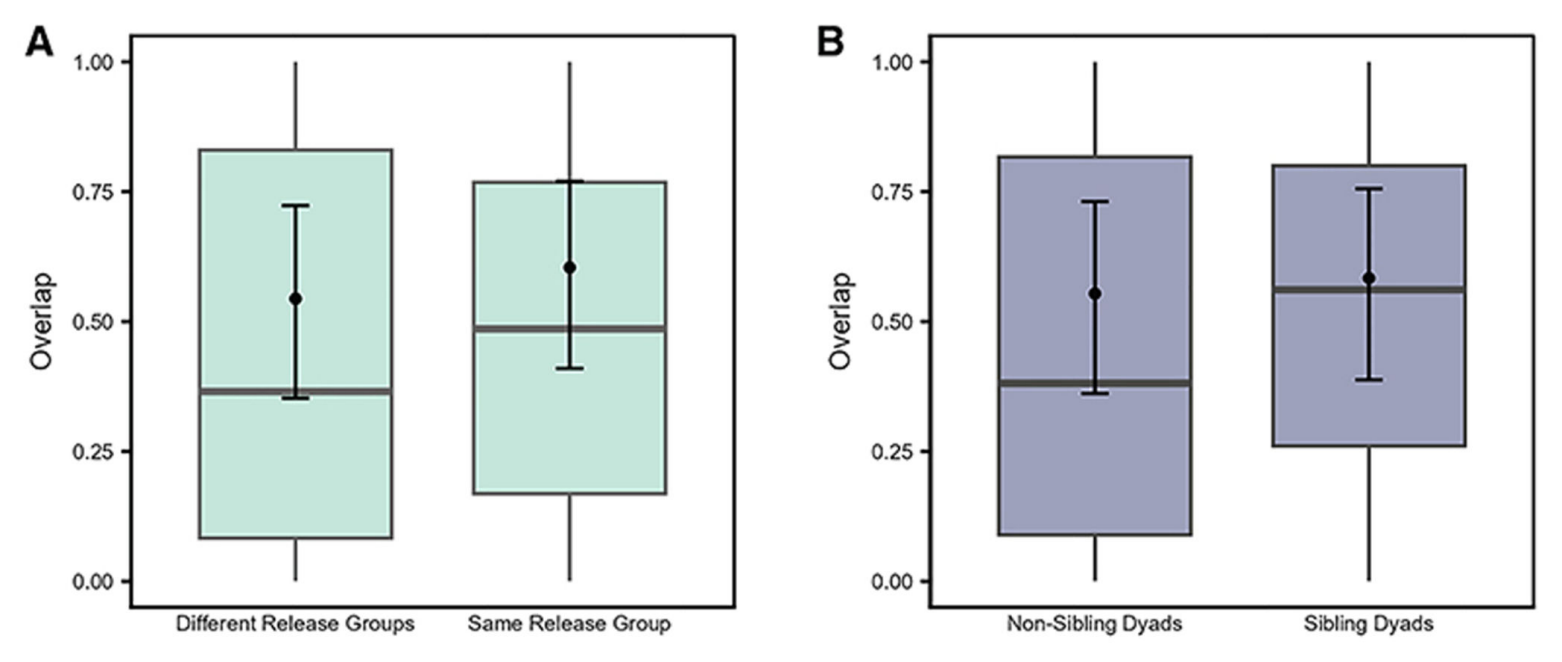
Familiar individuals are likely to overlap with each other more than unfamiliar individuals Predicted and observed range overlap for (A) dyads from different or same release groups, and (B) non-sibling and sibling dyads (QIII, reduced Model 2d). The *y*–axis represents the dyadic, directed response variable. Boxplots depict the median, 0.25 and 0.75 quantiles, with the whiskers indicating the range of the observations. Error bars indicate 95% confidence intervals around the estimated marginal means (black points).

The variation in the magnitude of range overlap (Model 2d) was not explained by the interaction between family size pair and overlap type (LRT: χ^2^ = 5.826, df = 3, *p* = 0.120), nor by the three-way interactions among overlap type and time with sibling status (LRT: χ^2^ = 0.257, df = 1, *p* = 0.612) or release group type (LRT: χ^2^ = 0.188, df = 1, *p* = 0.665). Hence, we fitted a reduced model that excluded all the test two- and three-way interactions, while retaining the main effects of family size pair, sibling status, and release group type, along with all the control predictors as in the full model. We detected a significant effect of release group type (LRT: χ^2^ = 29.060, df = 1, *p* < 0.001) and a trend for sibling status (LRT: χ^2^ = 3.090, df = 1, *p* = 0.079), but no effect of family size pair (Table S11). Irrespective of the overlap type (i.e., in the 50% or 95% ranges) and time, individuals from the same release group overlapped with each other significantly more than those from different release groups (Figure 10A), while sibling dyads showed a slightly higher overlap than non-sibling dyads (Figure 10B).

The variation in interindividual roosting distances was explained by the interaction of release group type and time (Model 3b, LRT: χ^2^ = 8.449, df = 1, *p* < 0.001), but not by the interaction of sibling status and time (LRT: χ^2^ = 0.331, df = 1, *p* = 0.565), nor by family size pair (LRT: χ^2^ = 2.733, df = 2, *p* = 0.255, Table S12).

**Figure 11:**
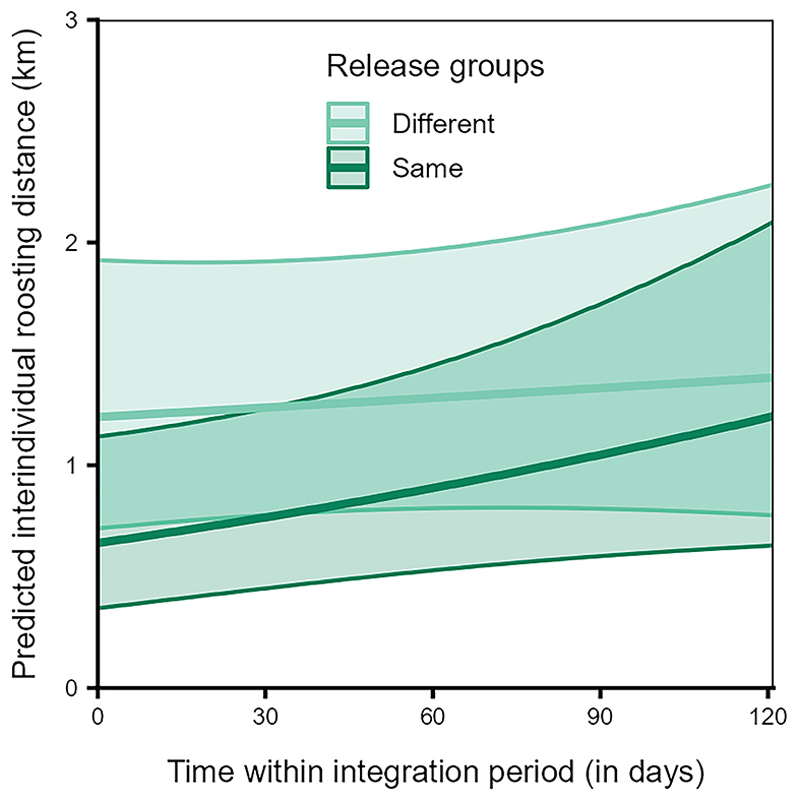
Individuals from the same release group roosted closer than those from different release groups Interindividual roosting distances during roosting for dyads from different (dark violet) or same release group (light blue) over time within the integration period (QIII, Model 3b). The *y*–axis depicts the back-transformed response variable (dyadic, undirected), which was statistically modeled as a continuous proportion. The ribbons indicate the 95% confidence intervals around the estimated adjusted predictions (thick line). See Figure S7 for the observed data distribution.

Overall, birds from the same release group roosted closer to each other than birds from different release groups (Figure 11). However, the increase in interindividual distances over time was steeper for dyads from the same release group as compared to dyads from different release groups (Figure 11).

## Discussion

This study presents a novel application of movement ecology to address open questions in behavioral ecology and social dynamics; specifically, how juvenile ravens integrate into non-breeder groups. We examined whether and how juvenile ravens gradually adjusted their spatial behavior to increase opportunities for encounters, align with local group activity patterns, and adapt to the local environmental and social contexts. To do so, we computed three metrics reflecting juveniles’ attraction to key flocking areas (metric 1) and to other conspecifics (metrics 2 and 3). Our results show that while juvenile ravens differed from older birds in their spatial behavior, their movement gradually aligned with those of the established non-breeder flock, thereby increasing the likelihood of spatial associations, essential for integration. Further, we found that spatio-temporal behavior of the birds varied based on differences in their rearing backgrounds (wild vs. captive parents). For captive-raised juveniles, familiarity from shared rearing environments, rather than early-life family size, predicted patterns of shared space use. These findings highlight the potential of fine-scale movement data to reveal hidden social processes during critical developmental transitions in wild animals.

### QI: Variations in spatial use patterns across age classes

We found that juveniles used the common locations more than older birds, especially at the start of the integration period (approximately coinciding with September of each year; Figure 3), suggesting that juveniles are indeed quickly attracted to areas known to be used by the local community. In contrast, the presence of older birds at these locations was lower at the start of the integration period. Still, it increased steeply toward the end of the integration period (approximately coinciding with December of each year). These patterns likely reflect the seasonal aspect of raven fission-fusion dynamics, with the colder season reflected toward the end of the integration period, leading to larger groups and a stronger reliance on anthropogenic food sources such as the Cumberland Wildpark.^51,52,58^ However, the brief temporal scope of our study prevents us from confirming these seasonal effects and limits our ability to interpret whether the juveniles over time conform to the seasonal area use patterns shown by older birds. Additionally, older birds that are present at our site in the summer and autumn tend to engage in mild conflicts with juveniles.^59^ Thus, we cannot exclude the alternate possibility that older birds may be avoiding the anthropogenic food sources upon the arrival of recently fledged juveniles, as was suggested in a study on the raven population in Greenland.^62^

We found that juveniles overlapped with the older birds in both their broader (95%) and core area (50%) ranges much more than older birds overlapped with juveniles (Figure 4). This could be a consequence of the juveniles’ ranges being much smaller than and largely covered by those of the older birds (95% range, juveniles: 11.31 ± 60.57 km^2^, sub-adults year 1: 43.45 ± 178.55 km^2^, sub-adults year 2: 39.11 ± 83.57 km^2^, adults: 49.50 ± 145.25 km^2^; 50% range, juveniles: 1.39 ± 9.05 km^2^, sub-adults year 1: 3.90 ± 16.11 km^2^, sub-adults year 2: 3.99 ± 9.83 km^2^, and adults: 5.38 ± 22.60 km^2^). Lower magnitudes of overlaps between the juvenile-juvenile dyads compared to older same-age dyads (sub-adults year 1 to year 1, sub-adults year 2 to year 2, and adults to adults; Figure 4) suggest that the juveniles may not have fully integrated with their peers. Looking closer, juveniles appear to stably maintain their core ranges with respect to each other, as indicated by hardly any temporal changes in the 50% range overlap. Further, the juveniles’ exploration ranges (95% ranges) may be increasing over time, resulting in an increase in the 95% range overlap, either due to convergence of the landscape explored by the juvenile dyads or due to co-exploration of areas by socially associated dyads. Similar developmental shifts in how peers share space and form associations have also been reported in yellow-bellied marmots,^26^ hihis (*Notiomystis cincta*),^63^ and great tits.^34^

Interestingly, we found that juveniles initially roosted closer to birds of all age classes. Unlike a recent study where most first-year crows (*Corvus corone*) were observed to roost individually,^64^ the juveniles in our study appear to immediately roost with conspecifics. Over the integration period, however, this proximity was only preserved with other juveniles, and to a lesser extent with the adults, while the roosting distances to the sub-adults increased gradually (Figure 5). Similarly, sleeping in clusters also decreases with age in black-and-gold howler monkeys (*Alouatta caraya*).^65^ We may interpret this as sub-adults avoiding juveniles, whereas adults are more tolerant toward them. This contradicts our expectation that juveniles would roost closer to all other birds as they socially integrate into the group over time. One possible explanation is that the daytime spatial use patterns of the juveniles (primarily reflecting shared feeding and socializing sites) may be quite different from their nighttime roosting patterns.^66^ Communal raven roosts function as information centers whereby individuals may gain knowledge about unpredictable food sources.^67,68^ Naive juveniles initially may rely on the social knowledge of food sources by roosting closer to older, experienced individuals.^67,69^ However, upon gaining access to this foraging knowledge, they may no longer be dependent on the association with older birds and hence may no longer need to roost together. Further, juveniles roosting close to same-aged peers may incur foraging benefits by employing “gang” foraging strategies at food sources close to the roosting sites,^70^ thus encouraging prolonged association and social bonding among the juveniles.

Analysis of spatial patterns in older dyads reveals that subadults (years 1 and 2) tend to have more core area overlap with other sub-adults, compared to their broader ranges. Additionally, the overlap shows minimal change over time, indicating stable spatio-temporal patterns among older, socially integrated individuals. Similar temporal stability in the broader range overlaps was also observed among adult dyads. However, their core range overlap markedly decreased (Figure 4). Older birds also initially roosted closer to each other, but these interindividual distances increased steeply over the integration period (approximately from early September to the end of December, Figure 5). The older dyads may be consistently congruent in their resource use landscape, reflected in their broader range overlaps. Nevertheless, the decrease in core range overlap and increase in interindividual roosting distances over time suggest that individuals increase their spacing from others as they grow older and potentially over the winter season. Unlike naive juveniles, experienced older ravens may afford to space out further and exploit more food sources during harsh winter months of higher foraging competition.^51^ Note that for ravens, the winter season (approximately December to February) is typically associated with pair bond formation, and thus older or mature ravens engaging in these behaviors may be favoring one or a few partners over others, while also actively seeking to distance themselves from potential competitors. Such shifts toward selective associations and/or reduced tolerance with age mirror findings in several mammalian taxa.^71–75^

### QII: Variations in spatial use patterns of individuals from wild or captive parents

The increased likelihood of juveniles from captive parents to use key flocking areas at the start of our study, compared to those from wild parents (Figure 6), resembles the observed differences between juveniles and older birds, respectively (Figure 3). Juveniles from captive parents used the common locations most often, followed by juveniles from wild parents, older birds from captive parents, and finally older birds from wild parents (Figure 7). Taken together, these findings support the notion that juveniles are indeed quickly attracted to areas used by the local flock, and this holds irrespective of their rearing background. Furthermore, the 50% and 95% range overlaps of the captive-wild dyads were much larger than the overlaps of the wild-captive dyads (Figure 8), likely resulting from captive-raised individuals on average having smaller ranges (95% range: 9.16 ± 99.51 km^2^, 50% range: 1.04 ± 16.75 km^2^, similar to the juveniles), covered by the larger ranges of the wild-raised individuals (95% range: 54.12 ± 144.32 km^2^, 50% range: 5.48 ± 15.00 km^2^). These similarities in the area use patterns of juveniles and captive-raised individuals (irrespective of age class) may reflect the lower vagrancy of these individuals, who may be limited in their experience and knowledge of food resources, inclining them to rely more on local, familiar areas.^56^ Further, the overlaps of captive-wild dyads showed an increasing trend over the integration period, whereas the wild-captive overlaps decreased, albeit to a lesser extent, with time. It could be that both groups reduce their ranges simultaneously over time (possibly due to reduced resource availability in the colder months) and converge on the use of the same foraging sites, resulting in a relative increase in the overlaps between captive-wild dyads.

Range overlaps among captive-captive dyads and wild-wild dyads suggest temporal stability in the shared space use among individuals of these two classes. The magnitude of overlap among captive-captive dyads, however, was higher than that among wild-wild dyads (Figure 8). Additionally, in the context of roosting, distances between dyads from captive parents were smaller. They increased less steeply over the integration period, as compared to distances between dyads from wild parents or mixed dyads (Figure 9). These patterns could be explained by higher familiarity (or kinship) among the captiveraised cohort as compared to the wild-raised cohort, potentially facilitating shared space use. Preferences toward familiar individuals and/or kin may likewise shape schooling behavior in Trinidadian guppies (*Poecilia reticulata*)^76^ or female-female social networks of wild giraffes (*Giraffa camelopardalis*).^77^ Alternatively, similarities in the captive rearing environment experienced by these individuals may drive behavioral/cognitive homophily, which has also been shown to influence social associations.^78–81^ Furthermore, individuals from wild parents likely represent a large pool of the population, covering a wide geographic range^50^ and vagrancy styles.^57^ Differences in how the individuals from captive and wild parents represent the population samples may thus also potentially explain the observed variation in interindividual roosting distances.

### QIII: Effect of early life social factors on the spatial use patterns of ravens from captive parents

We found no evidence for the effect of family size experienced during early life on any of our three spatial metrics, suggesting that it does not affect abilities for spatio-temporal behaviors needed for social integration. At the same time, we cannot exclude the possibility of delayed effects of the early-life social environment on social integration, such as maintaining an optimal degree of integration later in life. Other aspects of early environment that we did not measure in this study, such as the sex composition of the clutch, competition pressure within the family, or ecological pressures during rearing, may also later affect the movement patterns during integration. For instance, food supplementation affected the socio-spatial patterns of red kites (*Milvus milvus*) during dispersal.^82^ Note that most social environments vary over time and/or across life stages,^83^ which might be especially true for long-lived and wide-roaming species like ravens. Hence, individuals are likely to face the challenge of adapting to these social changes repeatedly throughout their life and thus may experience “integration” time and again.^16,60^ Our study reflects one aspect of the integration process, i.e., spatial movement patterns. However, early life conditions may affect other essential competences for integration later in life, such as individuals’ social skills.^84^ Indeed, individuals may also rely on other socio-cognitive skills to form social relationships and adjust their degrees of social integration, such as attention to social cues,^85^ to form and maintain social relationships. Recent work demonstrates that social attention develops across ontogeny in ravens and crows,^86^ potentially shaping how juveniles adjust their behavior during social integration.

Familiar individuals (i.e., from the same release groups) overlapped with each other more (independent of the core area or broader range, Figure 10A) and roosted closer to each other (Figure 11) than unfamiliar individuals (from different release groups). The fact that these individuals were held together in the peer groups for six weeks before release, may have allowed them to form social bonds, potentially leading to a greater number of close social partners, with whom they may prefer to share space.^87^ Furthermore, dyads from the same release groups increased in their interindividual roosting distances over time, while those from different release groups were stable (Figure 11). On one hand, these patterns may reflect seasonal effects in the roosting patterns, as was also found among dyads from different age classes and rearing backgrounds. On the other hand, individuals may space out from each other as they increase their social connections over time and become socially integrated in the group.^26^ Thus, as per our expectations and in line with previous studies,^19,21,22,26^ these results suggest that familiarity or prior social bonds drive the spatial usage patterns of ravens and may be key in facilitating social interactions or group cohesiveness, at least during the initial stages of social integration. Future studies integrating spatio-temporal data with social network analyses may be able to elucidate the stability of familiarity-based associations among juveniles and their influences on population-level social structure.^88^ More broadly, our results illustrate how established tools from movement ecology^89^ can be leveraged to uncover the social consequences of rearing history and familiarity, complementing network-based approaches that increasingly link movement and social dynamics across species.^88^

In conclusion, we have presented a novel quantitative assessment of social integration through movement patterns in wild birds. To explore the socio-ecological drivers of juvenile ravens’ movement during early social integration,^89^ we identified three spatial metrics that capture juveniles’ attraction to shared resource sites and other conspecifics as effective proxies for examining the spatial behavior that precedes the formation of social relationships. As hypothesized, we found that the spatial patterns of juveniles differ from those of older birds, gradually developing through time. We also found the spatial behavior of captive-reared individuals (irrespective of age class) to resemble that of juveniles, highlighting key considerations for reintroduction and conservation programs. The possibility that prior familiarity among juveniles may facilitate social integration provides insights into strategies for reintroduction programs. Although we did not detect any immediate effect of family size in early life, future research is needed to assess possible delayed effects or alternative influences on other socio-cognitive skills essential for social integration. Finally, given the limited existing research, we emphasize the need for studies integrating bio-logging, network analysis, and observational approaches to better understand the socio-cognitive and ecological mechanisms involved in the process of social integration in the wild.^90^

### Limitations of the study

We identify four key limitations of this study. First, not all individuals in our population are GPS-tagged. It is rarely feasible to tag and track entire populations. While this means that we may not be able to capture all fine-scale spatial associations, our relatively large sample of 156 GPS-tagged individuals nevertheless provided key insights into the movement patterns of the wild population in the context of social integration. Second, the short temporal window of the study limits our ability to fully grasp patterns spanning long periods, preventing us from determining whether the observed patterns in juveniles align with those of older birds over extended time frames, as observed in great tits.^34^ The shorter study period also does not allow us to examine possible delayed effects of early-life social conditions on the spatial usage metrics of individuals from captive parents. The third limitation concerns the inconsistency in the amount of GPS data collected over the study period, as solar-powered GPS backpacks may not get regularly charged during the winter days, typically characterized by heavy cloud cover and shorter daylight hours. Nevertheless, we have accounted for any variations in GPS samples when computing the GPS-based movement metrics and statistical analyses (see STAR Methods). Finally, we did not account for variation in the vagrancy of ravens from wild parents, who may have originated from other sub-populations along the Alps. In contrast, all the individuals from captive parents originated from one location—our study site where they were allowed into free flight. The impact of possible variations in spatial clustering arising from this on the trends we observed cannot be distinguished, especially in the case of the dyadic metrics.

## Resource Availability

### Materials availability

This study did not generate any materials.

### Lead contact

Requests for further information and resources should be directed to the lead contact, Awani Bapat (awani.bapat@univie.ac.at).

## Star★Methods

### Key Resources Table

**Table:** 

REAGENT or RESOURCE	SOURCE	IDENTIFIER
Deposited data		
Raw GPS transmitter data	Movebank Data Repository	206418248
Processed data and code	This paper	https://doi.org/10.5281/zenodo.17229149
Software and algorithms		
R versions 4.1.2 and 4.3.2	R Core Team	https://www.r-project.org/
QGIS version 3.42.0	QGIS Association	http://www.qgis.org
Other		
GPS Transmitters (OrniTrack-25 with elevated solar panels)	Ornitela UAB, Lithuania	https://www.ornitela.com/25g-transmitter

### Experimental Model And Study Participant Details

#### Subjects

This study was conducted on a free-flying population of common ravens in the Northern Austrian Alps. On average, 56% of the population is individually marked, comprising sexually immature juveniles (0–1 year), sub-adults in their first year (1–2 years), and subadults in their second year (2–3 years), and sexually mature adults (3 years and older). This study reports data from a total of 156 ravens (85 females, 71 males) of which 72 were wild-caught and 84 were captive-bred individuals.

The captive-bred individuals were hatched and raised by our captive breeding colony (with breeding pairs housed at the Konrad Lorenz Research Center, Grünau im Almtal, Upper Austria; Haidlhof Research Station, Bad Vöslau, Lower Austria; and Tiergarten/Zoo Schönbrunn, Vienna) and were allowed into free flight following a standardized procedure since 2018. As part of a project on social ontogeny, the brood size of the breeding pairs was manipulated to obtain small (one to two chicks) or large (three to four chicks) families. They stayed in their family groups until approximately 10 weeks post fledging (May to mid-July) and then were transferred to either two of the three non-breeder aviaries (old KLF: 2018–2021, GP Wood: 2017–2022, GP Moose: 2022, Figure 2) located at the Konrad Lorenz Research Center, Grünau im Almtal for an additional six weeks (mid-July to end August). During this peer-group phase, the offspring were exposed to behavioral and cognitive experiments. All birds were provided with food twice a day and water was available *ad libitum*. After six weeks in the captive peer groups, each individual was uniquely marked and equipped with a GPS logger (see below), before allowing them into free flight (typically, in the first week of September). All trapping, marking, and GPS tagging procedures are described under method details.

#### Study site

Our study site was the Cumberland Wildpark, Grünau im Almtal, Upper Austria, a zoo that is regularly used by the free-flying raven population for foraging, roosting, and socializing^58,91,92^ (Figure 2). Within the zoo, the ravens typically scavenge food from the enclosures of the wild boars (*Sus scrofa*), Eurasian wolves (*Canis lupus lupus*) and brown bears (*Ursus arctos*). They are also often sighted food-caching or socializing at the Przewalski’s horses (*Equus ferus Przewalski*), Alpine Chamois (*Rupicapra rupicapra)*, fallow deer *(Dama dama)*, and roe deer *(Capreolus capreolus)*.^57,58^ The zoo represents one of the many resources exploited by the ravens across the landscape – other unintentional human (e.g., compost sites, garbage dumps, ski huts, farms, restaurants, and hotels) and natural (e.g., carcasses, small vertebrates, grain, insects, and fruits) resources are also exploited at varying intensities.^51,52^ However, ravens are present in the zoo all year long, varying in numbers seasonally,^54,57,58,93^ thus proving it to be a reliable study site for long-term population monitoring. The exploitation of the zoo, alongside other anthropogenic resources, reflects a broader and long-standing pattern of raven association with human landscapes across a range of environments, where the human provisioning of food creates opportunities that these generalist scavengers readily exploit.^94,95^

#### Ethics

This research adheres to the ASAB/ABS Guidelines for the Use of Animals in Research. Permission for trapping, blood-sampling and GPS-tracking of wild ravens was granted by the Austrian Ministry for Education, Science and Research (approval numbers BMWF-66.006/0009-II/3b/2012, BMBWF-66.006/0015-V/3B/2018). Permission for the ringing and monitoring program of the Konrad Lorenz Research Center was authorized by the Central Administration of Upper Austria and permission for releasing the offspring of captive ravens into free flight was granted by the BH Gmunden (Ausnahmebewilligung §29 Oö. NSchG 2001). All methods are reported in accordance with the ARRIVE guidelines.

### Method Details

#### Trapping and marking procedures

Wild individuals were captured using drop-in traps, baited with meat and bread, located within the Cumberland Wildpark.^54,96^ Since 2008, about 600 individuals have been individually marked with a unique combination of color rings, colored patagial wing tags, and a numbered metal ring from Vogelwarte Radolfzell (2008–2017) or Austrian Ornithological Center (2017–2022). A sample of 50–200 μL of blood was taken from the ulnar vein to genetically determine the sex of the individuals. The age class of wild-caught individuals was determined based on mouth and feather coloration.^97^

#### GPS tagging

Between 2017 and 2022, a total of 173 individuals (representing all age classes and both sexes) were equipped with solar-powered GPS transmitters (OrniTrack-25 with elevated solar panels, Ornitela UAB, Lithuania; https://www.ornitela.com/25g-transmitter).^52^ All transmitters, at their weight once fully constructed, weighed 27g and never exceeded 3% of the bird’s bodyweight. The GPS transmitters were programmed to record data at battery charge-dependent frequencies, from sunrise to sunset, with one GPS fix recorded 6 h after sunset. During summer, the solar batteries often maintain a higher charge with more extended periods of daylight, resulting in higher sampling frequencies (i.e., typically ranging from 1s to 15min). Conversely, in winter, GPS locations are recorded every 1–2 h, with occasionally even longer gaps. Transmitter data are downloaded via GSM/GPRS/3G network, stored, and managed on Movebank, a database for animal tracking data. Over the years, on average 43% of the population was GPS tagged.

#### GPS-data processing

Data were downloaded from 156 ravens. However, due to computational differences of each metric, the operational sample sizes for each metric differ slightly (see below). The effective samples sizes for each metric can be found in Tables S1, S4, and S6. To examine the social integration of juvenile ravens, we focused on the first 122 days of movement data (hereafter, integration period), starting from the day on which the captive-bred individuals were allowed into free flight, across six years (2017–2022). We then computed three movement-based metrics at non-overlapping daily or biweekly (i.e., fortnightly) intervals (Figure 1) in R.^98^

#### Probability of using common locations in and around Cumberland Wildpark

To understand how individuals used different locations in and around the zoo, we calculated their probabilities of using four locations of interest for foraging and socializing within the zoo (i.e., horse, bears and wolves, wild boar, and deer enclosures), and the adjacent cliff (Figure 2). Note that the wild boar location also includes the chamois enclosure, and the deer location includes the fallow and roe deer enclosures. Around the center point selected for each location, we constructed a 100m circular buffer and looked at whether individual GPS fixes intersected with the buffer.^50^ Thus, we only focused on the 136 individuals that had intersected with any of the location buffers. We tallied the number of GPS fixes in versus out of the buffer daily using the R package ‘sf’.^99,100^ The probability of area use was estimated using daytime GPS fixes only (i.e., from sunrise to the start of sunset) to eliminate any bias of more numerous night-time points in winter months when day length is shorter.^101^

#### Range overlap

The individual range distributions were measured using daytime GPS fixes in biweekly intervals within the integration period. We filtered individuals with less than 10 fixes per interval to better estimate range sizes, thus resulting in 150 individuals. We estimated the range using the Kernel Density Estimate (KDE) method using the ‘amt’ package at 95% and 50% isopleth.^102^ KDE estimates the probability density of an individual’s locations across a landscape. The 95% KDE represents the spatial area enclosed by the contour lines containing 95% probability density, while the 50% KDE reflects more central and frequented (i.e., core) areas.^103^ Using the KDEs, we then calculated the proportion of overlap of one individual’s 95% and 50% ranges to another’s respective ranges, within each biweekly period.^102^

#### Interindividual roosting distance

We measured daily interindividual roosting distances using nighttime GPS fixes that our loggers were programmed to record 6 h after sunset. We isolated the single GPS fix at night, thus resulting in data from 152 individuals, and computed the Euclidean distances between combinations of the individuals using the function ‘distVincentyEllipsoid’ of the R package ‘geosphere’.^103^

### Quantification And Statistical Analysis

All statistical analyses were carried out in R.^98^ We fit Generalized Linear Mixed Models (GLMMs)^104^ with the function ‘glmmTMB’ of the package ‘glmmTMB’.^105^ Please refer to Note S1 for system information, R and glmmTMB version details, and R session information. We ran the model diagnostics using packages ‘DHARMa’ (version 0.4.7),^106^ ‘car’ (version 3.1-3)^107^ and custom functions by Roger Mundry.^108^

#### Model structure

##### Probability of using common locations (GPS Metric 1)

To analyze variation in the probability of using the common locations, we fitted a logistic GLMM,^109^ with the response variable as a two-column matrix of number of points inside and outside the corresponding location for each individual (Models 1a and 1b).

To examine variation among individuals of different age classes (QI) and origins (QII) over time, we included the main effects of age class, and origin, and the interactions of age class and time (number of days within the integration period), age class and origin, and origin and time as test fixed predictors (Model 1a). We included the main effects of sex, location, and time as control fixed predictors. We included the random intercepts of individual identity, release year, date, and observation number, along with all theoretically identifiable random slopes, to avoid pseudo replication.^110,111^

To examine the effect of family size of the individuals raised by captive parents (QIII), we analyzed a subset of the data with family size as the test fixed predictor (Model 1b). We included the main effects of sex, age class, location, time, and the Haversine distance between the release aviary and location, and the interaction term of age class and time, as control fixed predictors. We also included the same random intercepts as in Model 1a, with the addition of parent identity and release group, along with some of the theoretically identifiable random slopes (Note S2).

##### Range overlap (GPS Metric 2)

The response variable was a dyadic, directed, continuous proportion ranging from 0 to 1, with 45.54% of the observations as zeros. To avoid potential zero-inflation issues, we analyzed this variable in two parts: we first modeled the probability of an overlap occurring for each dyad (‘range overlap occurrence’) as a binary response variable, with values as 1 (non-zero overlap) or 0 (zero overlap) using a logistic GLMM^109^ (Models 2a and 2b). We then subset the data for all non-zero overlap cases and modeled the magnitude of overlap for each dyad (‘range overlap magnitude’) as a continuous proportion using a beta GLMM with a logit link function^109^ (Models 2c and 2d), after transforming the response to avoid values being exactly 0 or 1, using the following formula^112^:

(1) $$transformed\;response\;=\frac{\;response\;value\;\times \;(total\;number\;of\;observations\;-1)+0.5}{\;total\;number\;of\;observations\;}$$

To examine whether dyads based on age class (QI) and origin (QII) varied in the probability and magnitude of overlap in the 50% and 95% ranges over time, we included the main effects of age class and origin at the dyadic level (age class pair and origin pair respectively), overlap type (i.e., whether the overlap is in the 50% or 95% range), the two-way interactions of age class pair and overlap type, origin pair and overlap type, and overlap type and time (within integration period, measured as number of biweek), and the three-way interaction terms of age class pair, overlap type and time, and origin pair, overlap type and time as test fixed predictors (Models 2a and 2c). Additionally, we included sex at the dyadic level (sex pair) and time as control fixed predictors. To examine effect of the early-life social environment (QIII), we analyzed a subset of the data including only dyads of individuals from captive parents. We included the main effects of family size at the dyadic level (family size pair), whether they are siblings or not (sibling status), and whether they are from the same release group or not (release group type) as test fixed predictors, along with the two-way interactions of overlap type with family size pair, sibling status, and release group type, and of time with sibling status and release group type, and the three-way interactions of sibling status, overlap type and time, and release group type, overlap type and time as test predictors (Models 2b and 2d). We controlled for the main effects of sex pair, overlap type, age class pair, and time, and the three-way interaction of age class pair, overlap type and time (as above).

To avoid pseudo replication, all the above models included the random intercepts of dyad identity, identities of both individuals of the dyad, biweek identity, and release year, along with some of the theoretically identifiable random slopes (Note S2). Models 2b and 2d additionally included the random intercepts and (some) random slopes of the identities of the parents of both individuals of the dyad.

##### Interindividual roosting distance (GPS Metric 3)

The response variable of interindividual roosting distance was undirected, dyadic, and right-skewed, ranging from 0.21m to 219.98 km (Figure S8). The rare but extremely large distances could be due to some of the tagged individuals roosting far from our main study population described above. Looking into a few of these far-distance datapoints, we infer that this occurs either because they roost with a distant sub-group with fewer GPS-tagged individuals (as all our individuals are trapped and marked in the Cumberland Wildpark), or because they are territorial adults roosting away from non-breeder groups. In either case, including such large distances in our analyses is not relevant to our study focusing on the movement patterns of juveniles preceding their integration into the local population around the Wildpark. Thus, we subset our data to include interindividual roosting distances that were less than or equal to the 50% quantile (5.31 km) of our entire data. A circle of radius 5.31 km around our study site (Cumberland Wildpark) roughly covers the entire valley and can be assumed to include most of the roosting locations of our main study population. The resulting response variable was a continuous covariate with a natural lower bound of zero, but an artificial upper bound of 5311.62m. In order to statistically model this, we converted it into a continuous proportion of the maximum interindividual distance in our dataset by dividing all observations by 5311.62, resulting in a response ranging from 0 to 1. We then fitted GLMMs (Models 3a and 3b) with a beta family distribution and logit link function,^109^ after transforming the response to avoid exactly 0 and 1 values, using Equation 1 described above.

To examine variation among dyads based on age class (QI) and origin (QII), we included the main effects of age class pair and origin pair, and their two-way interactions with time (within the integration period, measured as number of days) as test fixed predictors (Model 3a). Similar as above, we controlled for the main effects of sex pair and time. To examine the effect of early social conditions (QIII), we again subset the data to include observations of dyads from captive parents only, including the main effects of family size pair, sibling status, and release group type, and the two-way interactions of sibling status and time, and release group type and time as the test fixed predictors (Model 3b). We controlled for the fixed effects of sex pair and the interaction of age class pair and time (as above). The random intercepts included for Models 3a and 3b were same as described above for Models 2a/2c and Models 2b/2d respectively, along with some of the corresponding random slopes.

#### Model diagnostics and inference

We checked for overdispersion for Models 1a and 1b using the function ‘testDispersion’ of package ‘DHARMa’,^106^ and for Models 2c, 2d, 3a, and 3b using the custom function ‘overdisp.test’ from Roger Mundry.^108^ We found that none of the models were overdispersed (Table S13). We tested for collinearity of fixed predictors using variance inflation factors (VIF, function ‘vif’ of R package ‘car’^107^), by fitting models excluding the respective interaction terms, which revealed no collinearity issues (overall max VIF = 1.779, Table S13). Finally, we visually examined the normal distribution of the best linear unbiased predictors (BLUPs) for all models (Figures S9–S16) and found that the BLUPs of Models 2a and 2b (Figures S11 and S12) had a very large x–axis range, suggesting that the estimated model coefficients may be unreliable and must be interpreted with extreme caution. In case of Model 2b, we additionally detected cases of complete separation for one level of the control fixed predictor of age class pair, where the observations of overlap occurrence for dyads of sub-adults year 1 were unbalanced, with no overlap (occurrence = 0) observed only four times, while an overlap (occurrence = 1) was observed 1,016 times. Thus, we did not interpret the results of this model further. We did not examine the model stability for our statistical models due to insufficient computational power.

To test the significance of the test fixed predictors of all the models, we compared the fit of the full models with that of the respective null models comprising of only the corresponding control fixed predictors (see Table 1) and random effects, using a likelihood ratio test.^113^ We tested the effects of individual fixed effects with a likelihood-ratio test by excluding each fixed effect term at a time using drop1 function. If the full-null model comparison was not significant, we did not further test for or interpret the effects of the individual test fixed predictors. The 95% confidence intervals for the model estimates were computed using the Wald method (function ‘confint’ of package ‘glmmTMB’^105^). In case of Model 2a, the large confidence intervals (Figures S1 and S2, Table S8) could be indicative of the unreliability of the estimated coefficients due to the large variation in the BLUPs.

#### Data visualization

All figures corresponding to statistical models and data were created using R packages ‘ggplot2’ (version 3.5.1),^114^ ‘ggeffects’ (version 1.7.0, function ‘ggpredict’),^115^ ‘gghalves’ (version 0.1.4)^116^ and ‘ggh4x’ (version 0.3.1).^117^ Figure 2 was created using QGIS (https://www.qgis.org). Raven silhouettes used in Figure 1 and the Graphical Abstract were obtained from PhyloPic (https://www.phylopic.org/).

## Supplementary Material

Supplemental information can be found online at https://doi.org/10.1016/j.isci.2025.114412.

Supplementary Material

## Figures and Tables

**Table 1:** Summary of fixed effects structures of each of the statistical models

	GPS metric 1: Use of common locations		GPS metric 2: Range overlap				GPS metric 3: Interindividual roosting distance
			Occurrence of range overlap		Magnitude of range overlap		
	Model 1a (binomial GLMM)		Model 2a (binomial GLMM)		Model 2c (beta GLMM)		Model 3a (beta GLMM)
Test fixed predictors (QI)	age class, age class: time in days		age class pair, age class pair: time in biweek periods, overlap type: age class pair, overlap type: time in biweek periods, overlap type: age class pair: time in biweek periods				age class pair, age class pair: time in days
Test fixed predictors (QII)	origin, origin: time in days, origin: age class		origin pair, origin pair: time in biweek periods, overlap type: origin pair, overlap type: origin pair: time in biweek periods				origin pair, origin pair: time in days
Control fixed predictors	sex, location, time in days		sex pair, time in biweek periods				sex pair, time in days
	**Model 1b (binomial GLMM)**		**Model 2b (binomial GLMM)**		**Model 2d (beta GLMM)**		**Model 3b (beta GLMM)**
Test fixed predictors (QIII)	family size		family size pair, overlap type: family size pair, sibling status, overlap type: sibling status, sibling status: time in biweek periods, overlap type: sibling status: time in biweek periods, release group type, overlap type: release group type: time in biweek periods, overlap type: release group type: time in biweek periods				family size pair, sibling status, sibling status: time in days, release group type, release group type: time in days
Control fixed predictors	sex, location, distance between release aviary and location, age class*time in days		sex pair, overlap type*age class pair*time in biweek periods				sex pair, age class pair*time in days

The statistical models correspond to the research questions addressing differences between age classes (QI) or rearing backgrounds (QII), and effects of early social environment of captive-reared individuals (QIII). Note that the data analyzed in Models 2c and 2d were subsets of the data used for Models 2a and 2b, respectively including only non-zero range overlap observations. Refer to STAR Methods for information on random effects structures and details of the statistical analyses.

## Data Availability

The GPS transmitter datasets generated and analyzed in this study are available in the Movebank Data Repository (study ID: 206418248); requests for access to which can be directed to the second correspondence, Thomas Bugnyar (thomas.bugnyar@univie.ac.at). The processed datasets used for statistical analyses have been deposited at https://doi.org/10.5281/zenodo.17229149 and are publicly available as of the date of publication. Accession numbers are listed in the key resources table. All original R code scripts and R data files corresponding to the analyses of the GPS metrics and statistical analyses have been deposited at https://doi.org/10.5281/zenodo.17229149 and are publicly available as of the date of publication. Any additional information required to reanalyze the data reported in this paper is available from the lead contact upon request.

## In brief

Wildlife behavior; zoology; ornithology; social integration; GPS telemetry; corvids; movement ecology; group living

## Highlights

- Juvenile ravens are quickly attracted to areas used by a nonbreeder flock
- Juveniles differed from older birds in how they share the area with conspecifics
- Spatio-temporal patterns of captive-reared juveniles are similar to those of wild
- Familiarity among captive-reared birds facilitated shared space use
