# Supplementary material for: Spatio-temporal patterns of juvenile common ravens integrating into a free-flying non-breeder flock
Source: iScience. Author manuscript; Available in PMC 2026 Feb 3. (PMC12818247; doi:10.1016/j.isci.2025.114412)
Supplement: Supplementary Material [file EMS212087-supplement-Supplementary_Material.zip › 1-s2.0-S2589004225026732-mmc1.pdf]

iScience, Volume *29*

Supplemental information

Spatio-temporal patterns of juvenile common ravens integrating into a free-flying non-breeder flock

Awani Bapat, Varalika Jain, Christian R. Blum, Palmyre H. Boucherie, Petra Sumasgutner, and Thomas Bugnyar

Supplemental Information

Table S1: Number of individuals and observations across age class, sex, and origin or family size for the probability of using common locations (Metric 1); related to Results and STAR Methods. Note that some individuals were sampled across multiple age classes.

		Number of Individuals					Number of Observations				
		Age Class					Age Class				
		0-1	1-2	2-3	3+	Total	0-1	1-2	2-3	3+	Total
Model 1a (full data set) - Distribution across Origin, Sex and Age class											
**Wild-caught**	**f**	12	13	6	9	40	1557	2720	1903	2068	8248
	**m**	23	19	15	20	77	596	1496	1012	917	4021
**Captive-bred**	**f**	36	15	8	4	63	3421	2421	607	1138	7587
	**m**	33	10	4	2	49	3669	3340	1372	2622	11003
**Total**		104	57	33	35	229	9243	9977	4894	6745	30859
Model 1b (data subset for captive-bred individuals only) - Distribution across Family Size, Sex and Age class											
**Large Family**	**f**	18	3	0	0	21	1519	916	0	0	2435
	**m**	22	9	5	3	39	2293	2084	1023	1828	7228
**Small Family**	**f**	15	6	3	1	25	1902	1208	318	91	3519
	**m**	14	5	2	0	21	1376	996	88	0	2460
**Total**		69	23	10	4	106	7090	5204	1429	1919	15642

	Female	Male	Total	Female	Male	Total
Model 1a (full data set) - Distribution across Origin and Sex						
**Wild-caught**	43	24	67	8248	4021	12269
**Captive-bred**	34	37	71	7587	11003	18590
**Total**	77	61	138	15835	15024	30859
Model 1b (data subset for captive-bred individuals only) - Distribution across Family Size and Sex						
**Large Family**	18	22	40	2435	7228	9663
**Small Family**	15	14	29	3519	2460	5979
**Total**	33	36	69	5954	9688	15642

Table S2: Number of dyads and observations across age class pair, sex pair, origin pair, family size pair, sibling status, and release group type for range overlap occurrence (Metric 2: models 2a and 2b); related to Results and STAR Methods. Note that some dyads were sampled across multiple age classes.

Model 2a (full-data set)		
	Number of Dyads	Number of Observations
**Total**	5154	139260
**Age Class Pair**		
0-1 & 0-1	941	11336
0-1 & 1-2	1212	10442
0-1 & 2-3	745	6202
0-1 & 3+	1348	10382
1-2 & 0-1	1212	10442
1-2 & 1-2	425	10904
1-2 & 2-3	527	6552
1-2 & 3+	727	8350
2-3 & 0-1	745	6202
2-3 & 1-2	527	6552
2-3 & 2-3	207	5016
2-3 & 3+	652	7334
3+ & 0-1	1348	10382
3+ & 1-2	727	8350
3+ & 2-3	652	7334
3+ & 3+	495	13480
**Sex Pair**		
Female-Female	1577	43732
Female-Male	2569	34856
Male-Female	2569	34856
Male-Male	1007	25816
**Origin Pair**		
Wild-caught & Wild-caught	1427	48548
Wild-caught & Captive-bred	2481	33130
Captive-bred & Wild-caught	2481	33130
Captive-bred & Captive-bred	1246	24452

Model 2b (data subset for captive-bred-captive-bred pairs)		
	Number of Dyads	Number of Observations
**Total**	1112	19756
**Age Class Pair**		
0-1 & 0-1	501	5808
0-1 & 1-2	326	2692
0-1 & 2-3	156	1112
0-1 & 3+	129	1074
1-2 & 0-1	326	2692
1-2 & 1-2	43	1020
1-2 & 2-3	53	560
1-2 & 3+	41	564
2-3 & 0-1	156	1112
2-3 & 1-2	53	560
2-3 & 2-3	10	208
2-3 & 3+	23	254
3+ & 0-1	129	1074
3+ & 1-2	41	564
3+ & 2-3	23	254
3+ & 3+	5	208
**Sex Pair**		
Female-Female	201	2820
Female-Male	572	4930
Male-Female	572	4930
Male-Male	339	7076
**Family size Pair**		
Large-Large	402	6356
Large-Small	527	4760
Small-Large	527	4760
Small-Small	183	3880
**Siblings**	59	1064
**Non-siblings**	1053	18692
**Same release group**	253	9368

**Different release groups**	859	10388

Table S3: Number of dyads and observations across age class pair, sex pair, origin pair, family size pair, sibling status, and release group type for range overlap magnitude (Metric 2 - models 2c and 2d); related to Results and STAR Methods. The data represents a subset of the data in Table S2 including only observations with range overlap greater than 0. Note that some dyads were sampled across multiple age classes.

Model 2c (full-data set)		
	Number of Dyads	Number of Observations
**Total**	4043	75840
**Age Class Pair**		
0-1 & 0-1	807	7654
0-1 & 1-2	1003	6521
0-1 & 2-3	531	3523
0-1 & 3+	969	5689
1-2 & 0-1	1003	6521
1-2 & 1-2	328	6248
1-2 & 2-3	375	3265
1-2 & 3+	544	4396
2-3 & 0-1	531	3523
2-3 & 1-2	375	3265
2-3 & 2-3	130	2332
2-3 & 3+	420	3464
3+ & 0-1	969	5689
3+ & 1-2	544	4396
3+ & 2-3	420	3464
3+ & 3+	329	5890
**Sex Pair**		
Female-Female	1258	22206
Female-Male	2016	19093
Male-Female	2016	19093
Male-Male	769	15448
**Origin Pair**		
Wild-caught & Wild-caught	1027	19456
Wild-caught & Captive-bred	1876	17953
Captive-bred & Wild-caught	1876	17953
Captive-bred & Captive-bred	1140	20478

Model 2d (data subset for captive-bred-captive-bred pairs)		
	Number of Dyads	Number of Observations
**Total**	1012	16266
**Age Class Pair**		
0-1 & 0-1	430	4450
0-1 & 1-2	309	2332
0-1 & 2-3	146	864
0-1 & 3+	126	857
1-2 & 0-1	309	2332
1-2 & 1-2	43	1016
1-2 & 2-3	53	493
1-2 & 3+	41	505
2-3 & 0-1	146	864
2-3 & 1-2	53	493
2-3 & 2-3	10	148
2-3 & 3+	23	194
3+ & 0-1	126	857
3+ & 1-2	41	505
3+ & 2-3	23	194
3+ & 3+	5	162
**Sex Pair**		
Female-Female	189	2284
Female-Male	517	4017
Male-Female	517	4017
Male-Male	306	5948
**Family size Pair**		
Large-Large	364	5284
Large-Small	478	3917
Small-Large	478	3917
Small-Small	170	3148
**Siblings**	50	938
**Non-siblings**	962	15328
**Same release group**	239	3104

**Different release groups**	773	13162

Table S4: Number of unique individuals of different sexes, and origin or family size included in the dyads for range overlap (Metric 2); related to Results and STAR Methods.

	Model 2a & 2c (full-data set)			Model 2b & 2d (data subset for captive-bred − captive-bred pairs)		
	**Wild-caught**	**Captive-bred**	**Total**	**Large Family**	**Small Family**	**Total**
**Female**	44	39	83	22	16	39
**Male**	27	40	67	23	16	40
**Total**	71	79	150	45	32	77

Table S5: Number of dyads and observations across age class pair, sex pair, origin pair, family size pair, sibling status, and release group type for interindividual roosting distances (Metric 3); related to Results and STAR Methods. Note that some dyads were sampled across multiple age classes.

Model 3a (full-data set)		
	Number of Dyads	Number of Observations
**Total**	4021	191542
**Age Class Pair**		
0-1 & 0-1	852	22454
0-1 & 1-2	999	36658
0-1 & 2-3	534	18420
0-1 & 3+	962	29600
1-2 & 1-2	317	15884
1-2 & 2-3	360	15684
1-2 & 3+	529	20774
2-3 & 2-3	118	4853
2-3 & 3+	385	14637
3+ & 3+	302	12578
**Sex Pair**		
Female-Female	1145	52565
Female-Male	2028	97452
Male-Male	848	41525
**Origin Pair**		
Wild-caught & Wild-caught	964	39431
Wild-caught & Captive-bred	1852	91095
Captive-bred & Captive-bred	1205	61016

Model 3b (data subset for captive-bred − captive-bred pairs)		
	Number of Dyads	Number of Observations
**Total**	1073	47044
**Age Class Pair**		
0-1 & 0-1	472	13444
0-1 & 1-2	326	14195
0-1 & 2-3	154	4034
0-1 & 3+	121	5436
1-2 & 1-2	43	2903
1-2 & 2-3	53	2319
1-2 & 3+	41	3013
2-3 & 2-3	10	301
2-3 & 3+	23	875
3+ & 3+	5	524
**Sex Pair**		
Female-Female	168	6151
Female-Male	537	23201
Male-Male	368	17692
**Family size Pair**		
Large-Large	397	15338
Large-Small	490	22480
Small-Small	186	9226
**Siblings**	56	2886
**Non-siblings**	1017	44158
**Same release group**	242	9118
**Different release groups**	831	37926

Table S6: Number of unique individuals of different sexes, and origin or family size included in the dyads for interindividual roosting distances (Metric 3); related to Results and STAR Methods.

	Model 3a (full-data set)			Model 3b (data subset for captive-bred − captive-bred pairs)		
	**Wild-caught**	**Captive-bred**	**Total**	**Large Family**	**Small Family**	**Total**
**Female**	43	37	80	19	17	36
**Male**	27	43	70	26	16	42
**Total**	70	80	150	45	33	78

Table S7: Model results for effect of interactions of age class with time within integration period (in days, QI), and origin with age class and time (QII) on the probability of using common locations (Model 1a); related to Results, Figures 3, 6, and 7. Test fixed predictors are indicated in bold. The χ^2, df, and p-value correspond to the corresponding likelihood ratio tests. Significance codes: * <0.05, ** < 0.01, *** <0.001. Reference categories are "female" for Sex; "bear-wolves" for Location; "Juvenile" for Age class; and "Wild-caught" for Origin. [1]This covariate was z-transformed before including in the model.

Model 1a: Probability of using common zoo locations (binomial GLMM)							
**Term**	**Estimate**	**SE**	**95% CL_{lower}**	**95% CL_{upper}**	**χ^2**	**df**	**p-value**
(Intercept)	-0.723	0.235	-1.184	-0.262			
Sex Male	0.215	0.132	-0.043	0.474	2.634	1	0.105
Location Cliff	-0.864	0.171	-1.199	-0.529	25.991	4	<0.001***
Location Deer	-0.888	0.250	-1.379	-0.398			
Location Horse	-0.449	0.217	-0.875	-0.024			
Location Wildboar	0.344	0.203	-0.055	0.743			
Age class Sub-adult Year 1	-1.015	0.201	-1.409	-0.621			
Age class Sub-adult Year 2	-1.041	0.301	-1.631	-0.452			
Age class Adult	-0.504	0.300	-1.092	0.084			
Origin Captive-bred	0.104	0.239	-0.364	0.572			
Number days within integration period[1]	0.200	0.078	0.047	0.353			
**Age class Sub-adult Year 1 : Origin Captive-bred**	**0.536**	**0.050**	**0.437**	**0.634**	**172.740**	**3**	**<0.001*** **
**Age class Sub-adult Year 2 : Origin Captive-bred**	**0.762**	**0.069**	**0.627**	**0.897**			
**Age class Adult : Origin Captive-bred**	**0.320**	**0.077**	**0.169**	**0.471**			
**Age class Sub-adult Year 1 : Number days within integration period[1]**	**0.282**	**0.088**	**0.108**	**0.455**	**22.102**	**3**	**<0.001*** **
**Age class Sub-adult Year 2 : Number days within integration period[1]**	**0.451**	**0.089**	**0.276**	**0.626**			
**Age class Adult : Number days within integration period[1]**	**0.461**	**0.107**	**0.252**	**0.670**			
**Origin Captive-bred : Number days within integration period[1]**	**-0.143**	**0.066**	**-0.271**	**-0.014**	**3.862**	**1**	**0.049***

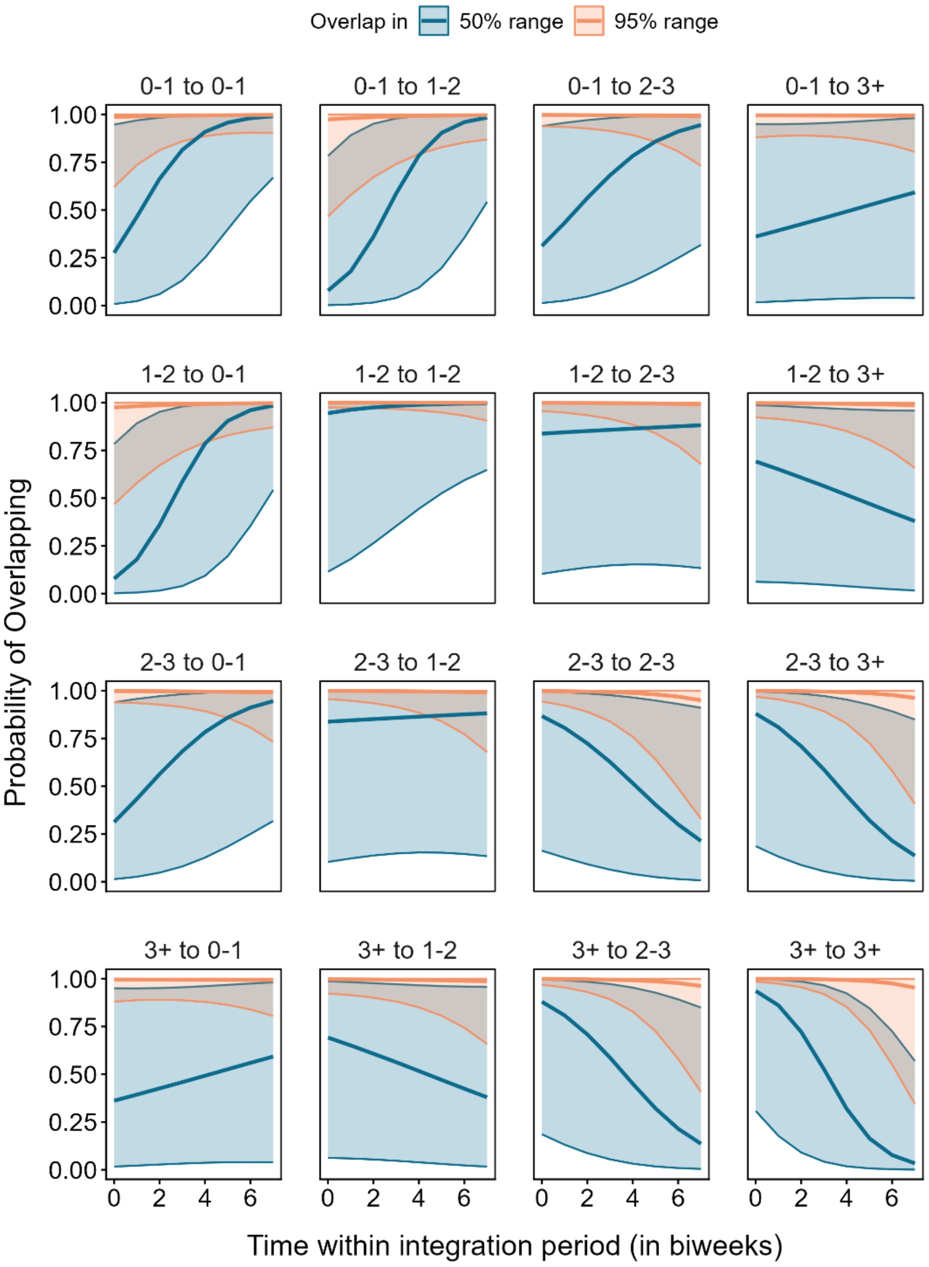

Figure S1: Probability of overlapping in the 50% (dark blue) and 95% (light orange) ranges (response variable 2) for dyads of individuals of different age classes (0-1: juveniles, 1-2: sub-adults year 1, 2-3: sub-adults year 2, 3+: adults) over time within the integration period (QI, Model 2a); related to Results and STAR Methods. The y-axis represents the dyadic, directed response variable. The ribbons indicate the 95% confidence intervals around the estimated adjusted predictions (thick line).

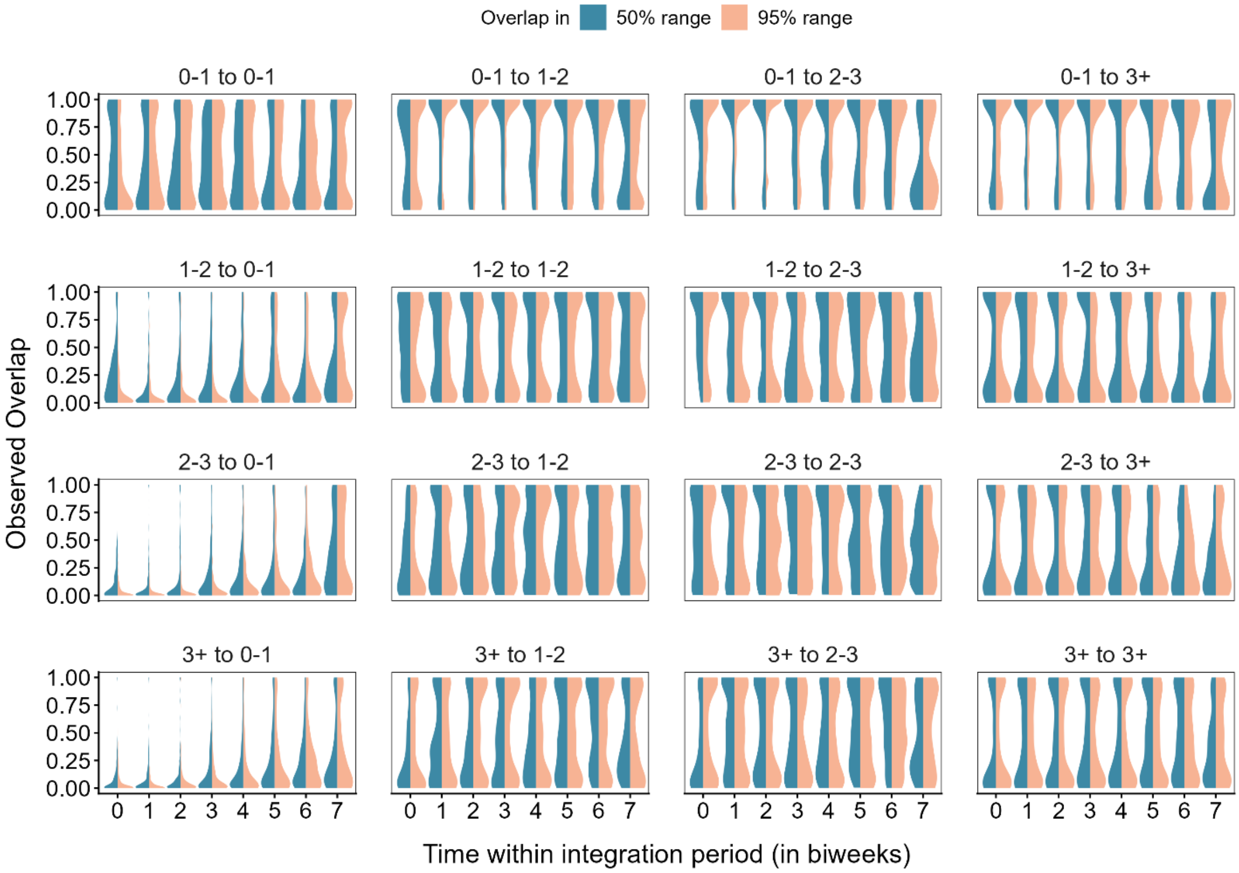

Figure S2: Observed data distribution for overlap of 50% (dark blue) and 95% (light orange) ranges (response variable 2) for dyads of individuals of different age classes (0-1: juveniles, 1-2: sub-adults year 1, 2-3: sub-adults year 2, 3+: adults) over time within the integration period; related to Figure 4. The y-axis represents the dyadic, directed response variable. The half violin plots indicate the density of the observations over the range of the response variables.

Table S10: Model results for effect of interactions of age class pair (QI) and origin pair (QII) with time within the integration period (in days) on the interindividual roosting distance (Model 3a); related to Results, Figures 5 and 9. Test fixed predictors are indicated in bold. The χ^2, df, and p-value correspond to the corresponding likelihood ratio tests. Significance codes: * <0.05, ** < 0.01, *** <0.001. Reference categories are "female-female" for Sex pair; "Adult-Adult" for Age class pair; and "Captive-bred-Captive-bred" for Origin pair. [1]This covariate was z-transformed before including in the model.

Model 3a: Inter-individual roosting distance (beta GLMM)							
**Term**	**Estimate**	**SE**	**95% CL_{lower}**	**95% CL_{upper}**	**χ^2**	**df**	**p-value**
(Intercept)	-1.033	0.310	-1.641	-0.424			
Sex pair female-male	-0.074	0.147	-0.361	0.214	0.275	2	0.872
Sex pair male-male	0.004	0.244	-0.474	0.481			
Number days within integration period[1]	0.219	0.086	0.049	0.388			
Age class pair Juvenile-Adult	-0.436	0.132	-0.696	-0.177			
Age class pair Juvenile-Juvenile	-1.102	0.273	-1.637	-0.568			
Age class pair Juvenile-Sub-adult Year 1	-0.520	0.264	-1.038	-0.002			
Age class pair Juvenile-Sub-adult Year 2	-0.103	0.272	-0.637	0.430			
Age class pair Sub-adult Year 1-Adult	-0.265	0.185	-0.627	0.097			
Age class pair Sub-adult Year 1-Sub-adult Year 1	0.046	0.339	-0.619	0.711			
Age class pair Sub-adult Year 1-Sub-adult Year 2	0.117	0.243	-0.359	0.593			
Age class pair Sub-adult Year 2-Adult	-0.074	0.161	-0.389	0.242			
Age class pair Sub-adult Year 2-Sub-adult Year 2	0.280	0.273	-0.255	0.816			
**Age class pair Juvenile-Adult : Number days within integration period[1]**	**-0.285**	**0.037**	**-0.357**	**-0.213**	**546.707**	**9**	**<0.001*** **
**Age class pair Juvenile-Juvenile : Number days within integration period[1]**	**-0.327**	**0.052**	**-0.429**	**-0.224**			
**Age class pair Juvenile-Sub-adult Year 1 : Number days within integration period[1]**	**-0.121**	**0.046**	**-0.212**	**-0.030**			
**Age class pair Juvenile-Sub-adult Year 2 : Number days within integration period[1]**	**-0.121**	**0.043**	**-0.206**	**-0.036**			
**Age class pair Sub-adult Year 1-Adult : Number days within integration period[1]**	**-0.026**	**0.029**	**-0.084**	**0.032**			

**Age class pair Sub-adult Year 1-Sub-adult Year 1 : Number days within integration period[1]**	**0.183**	**0.044**	**0.097**	**0.269**			
**Age class pair Sub-adult Year 1-Sub-adult Year 2 : Number days within integration period[1]**	**0.085**	**0.036**	**0.014**	**0.157**			
**Age class pair Sub-adult Year 2-Adult : Number days within integration period[1]**	**0.039**	**0.024**	**-0.007**	**0.085**			
**Age class pair Sub-adult Year 2-Sub-adult Year 2 : Number days within integration period[1]**	**0.141**	**0.043**	**0.056**	**0.226**			
Origin pair Wild-caught-Captive-bred	0.297	0.117	0.067	0.527			
Origin pair Wild-caught-Wild-caught	0.008	0.219	-0.423	0.438			
**Origin pair Wild-caught-Captive-bred : Number days within integration period[1]**	**0.171**	**0.047**	**0.079**	**0.264**	**25.794**	**2**	**<0.001*** **
**Origin pair Wild-caught-Wild-caught : Number days within integration period[1]**	**0.124**	**0.086**	**-0.045**	**0.293**			

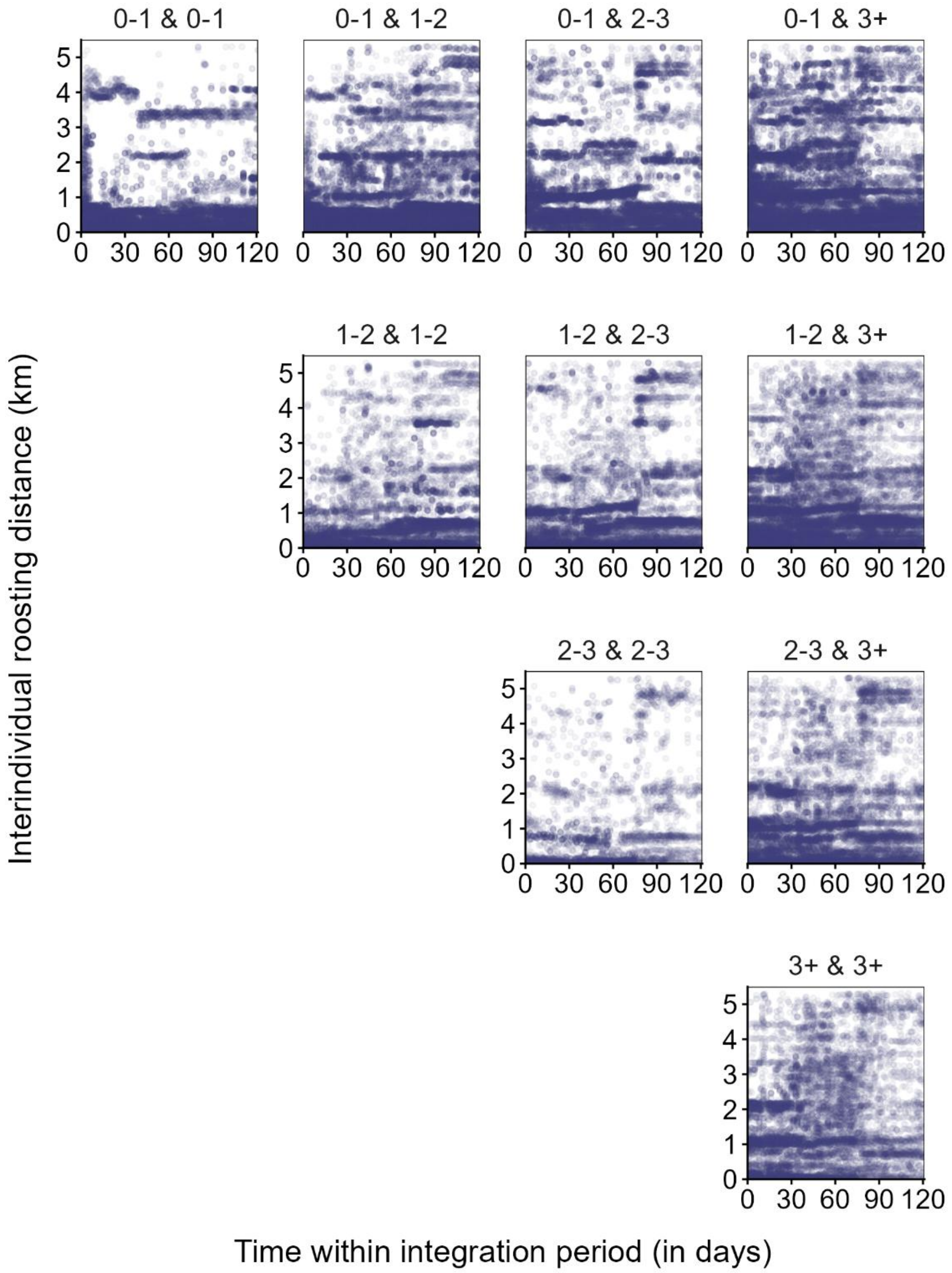

Figure S3: Observed data distribution for interindividual roosting distances (response variable 3) for dyads of individuals of different age classes (0-1: juveniles, 1-2: sub-adults year 1, 2-3: sub-adults year 2, 3+: adults) over time within the integration period; related to Figure 5. The topmost row depicts observations of all dyads of which at least one individual is a juvenile.

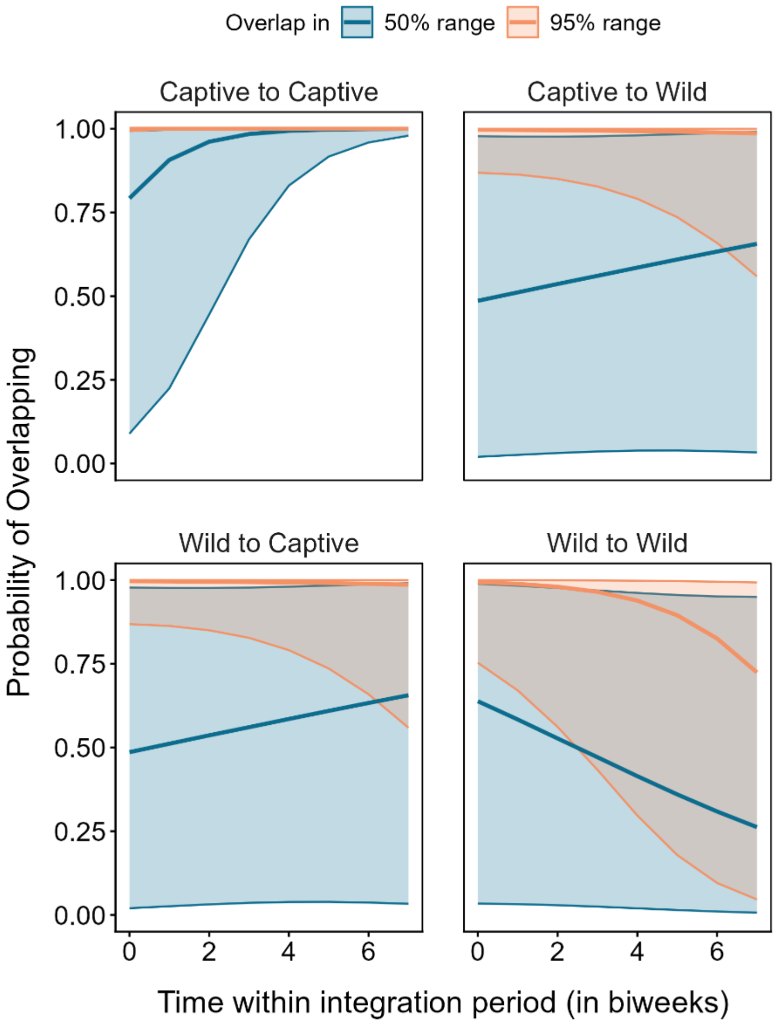

Figure S4: Probability of overlapping in the 50% (dark blue) and 95% (light orange) ranges (response variable 2) for dyads of individuals of different origin (from captive or wild parents) over time within the integration period (QII, Model 2a); related to Results and STAR Methods. The y-axis represents the dyadic, directed response variable. The ribbons indicate the 95% confidence intervals around the estimated adjusted predictions (thick line).

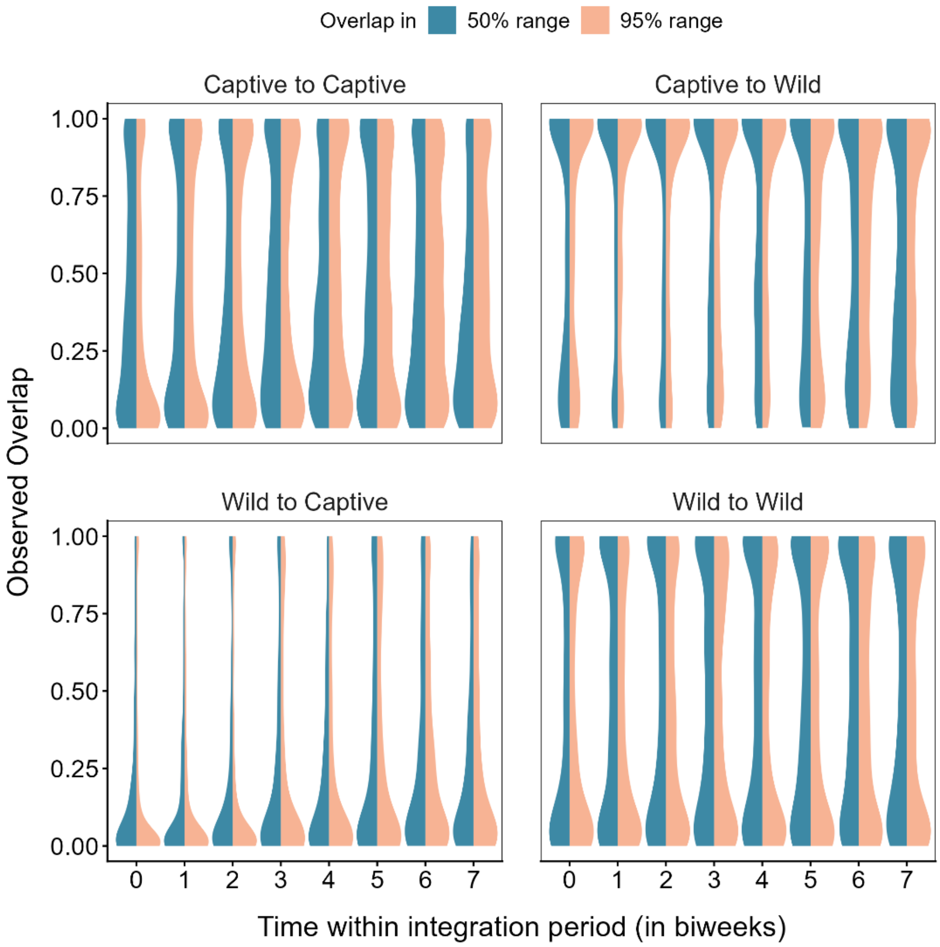

Figure S5: Observed data distribution for overlap of 50% (dark blue) and 95% (light orange) ranges (response variable 2) for dyads of individuals of different origin (from captive or wild parents) over time within the integration period; related to Figure 8. The y-axis represents the dyadic, directed response variable. The half violin plots indicate the density of the observations over the range of the response variable.

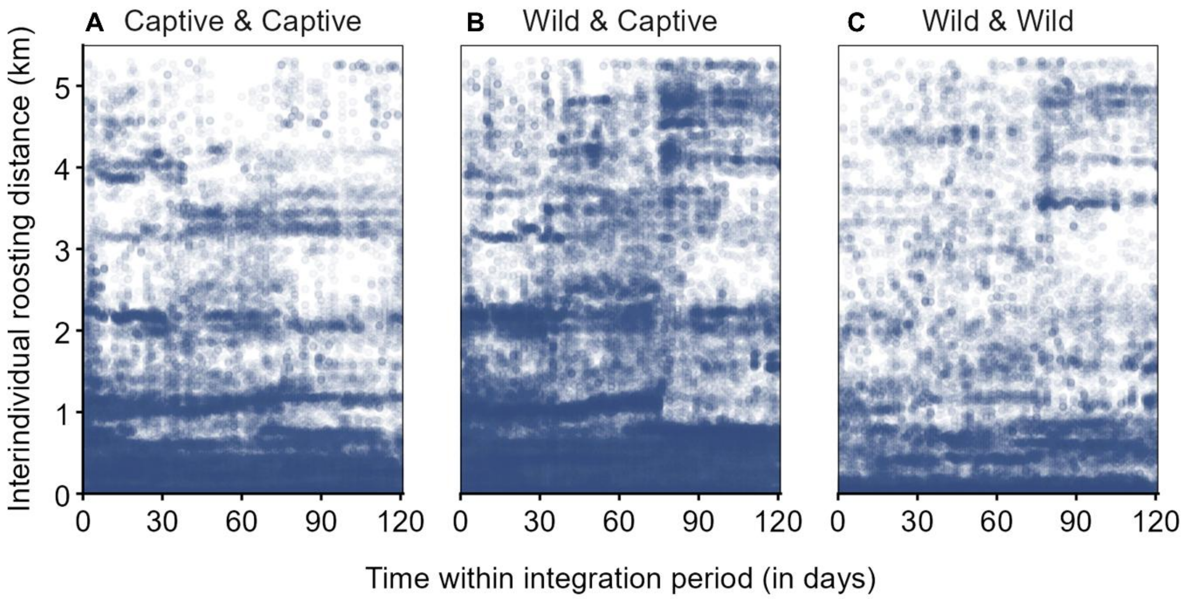

Figure S6: Observed data distribution for interindividual roosting distances (response variable 3) for dyads of individuals of different origin (**A**: both individuals from captive parents, **B**: individuals from captive parents and wild parents, **C**: both individuals from wild parents) over time within the integration period; related to Figure 9.

Table S11: Model results for effect of family size pair, sibling status, and release group type (QIII) on the magnitude of range overlap (reduced Model 2d); related to Results and Figure 10. Test fixed predictors are indicated in bold. Reference categories are "female-female" for Sex pair, "Adult-Adult" for Age class pair, "Large-Large" for Family size pair, "Different" for Release group type, and "No" for Sibling status. [1]This covariate was z-transformed before including in the model.

Reduced Model 2d: Magnitude of range overlap (beta GLMM)							
**Term**	**Estimate**	**SE**	**95% CL_{lower}**	**95% CL_{upper}**	**χ^2**	**df**	**p-value**
(Intercept)	1.478	0.609	0.284	2.672			
Sex pair female-male	0.049	0.260	-0.460	0.559			
Sex pair male-female	0.000	0.225	-0.442	0.442			
Sex pair male-male	0.091	0.342	-0.579	0.761			
Overlap type Overlap_95	-0.355	0.293	-0.930	0.220			
Overlap type Overlap_95 : Number biweek within integration period[1]	0.140	0.181	-0.215	0.495			
Number biweek within integration period[1]	-0.034	0.200	-0.425	0.357			
Age class pair Adult-Juvenile	-3.860	0.273	-4.395	-3.324			
Age class pair Adult-Sub-adult Year 1	-1.970	0.240	-2.441	-1.500			
Age class pair Adult-Sub-adult Year 2	-0.775	0.225	-1.215	-0.335			
Age class pair Juvenile-Adult	2.004	0.266	1.484	2.525			
Age class pair Juvenile-Juvenile	-1.775	0.341	-2.443	-1.107			
Age class pair Juvenile-Sub-adult Year 1	0.019	0.312	-0.592	0.631			
Age class pair Juvenile-Sub-adult Year 2	1.099	0.290	0.531	1.667			
Age class pair Sub-adult Year 1-Adult	1.249	0.233	0.792	1.707			
Age class pair Sub-adult Year 1-Juvenile	-2.864	0.314	-3.480	-2.249			
Age class pair Sub-adult Year 1-Sub-adult Year 1	-0.698	0.285	-1.256	-0.140			
Age class pair Sub-adult Year 1-Sub-adult Year 2	0.385	0.265	-0.134	0.904			
Age class pair Sub-adult Year 2-Adult	0.249	0.226	-0.194	0.692			
Age class pair Sub-adult Year 2-Juvenile	-3.340	0.294	-3.916	-2.764			
Age class pair Sub-adult Year 2-Sub-adult Year 1	-1.477	0.268	-2.002	-0.952			
Age class pair Sub-adult Year 2-Sub-adult Year 2	-0.347	0.266	-0.868	0.173			
Age class pair Adult-Juvenile : Overlap type Overlap_95	0.429	0.230	-0.021	0.880			
Age class pair Adult-Sub-adult Year 1 : Overlap type Overlap_95	0.395	0.231	-0.057	0.848			
Age class pair Adult-Sub-adult Year 2 : Overlap type Overlap_95	0.008	0.251	-0.484	0.499			

Age class pair Juvenile-Adult : Overlap type Overlap_95	0.099	0.250	-0.391	0.589			
Age class pair Juvenile-Juvenile : Overlap type Overlap_95	0.244	0.253	-0.252	0.741			
Age class pair Juvenile-Sub-adult Year 1 : Overlap type Overlap_95	0.523	0.251	0.030	1.015			
Age class pair Juvenile-Sub-adult Year 2 : Overlap type Overlap_95	0.313	0.254	-0.184	0.810			
Age class pair Sub-adult Year 1-Adult : Overlap type Overlap_95	-0.476	0.242	-0.950	-0.002			
Age class pair Sub-adult Year 1-Juvenile : Overlap type Overlap_95	0.048	0.242	-0.426	0.523			
Age class pair Sub-adult Year 1-Sub-adult Year 1 : Overlap type Overlap_95	-0.154	0.243	-0.630	0.323			
Age class pair Sub-adult Year 1-Sub-adult Year 2 : Overlap type Overlap_95	-0.332	0.247	-0.816	0.151			
Age class pair Sub-adult Year 2-Adult : Overlap type Overlap_95	-0.202	0.257	-0.706	0.303			
Age class pair Sub-adult Year 2-Juvenile : Overlap type Overlap_95	0.258	0.238	-0.209	0.724			
Age class pair Sub-adult Year 2-Sub-adult Year 1 : Overlap type Overlap_95	0.355	0.240	-0.116	0.826			
Age class pair Sub-adult Year 2-Sub-adult Year 2 : Overlap type Overlap_95	-0.219	0.283	-0.774	0.335			
Age class pair Adult-Juvenile : Number biweek within integration period[1]	0.059	0.176	-0.286	0.403			
Age class pair Adult-Sub-adult Year 1 : Number biweek within integration period[1]	0.117	0.172	-0.220	0.455			
Age class pair Adult-Sub-adult Year 2 : Number biweek within integration period[1]	0.050	0.188	-0.319	0.418			
Age class pair Juvenile-Adult : Number biweek within integration period[1]	0.113	0.173	-0.227	0.453			
Age class pair Juvenile-Juvenile : Number biweek within integration period[1]	-0.075	0.167	-0.402	0.252			
Age class pair Juvenile-Sub-adult Year 1 : Number biweek within integration period[1]	-0.063	0.165	-0.387	0.262			
Age class pair Juvenile-Sub-adult Year 2 : Number biweek within integration period[1]	0.032	0.173	-0.306	0.371			
Age class pair Sub-adult Year 1-Adult : Number biweek within integration period[1]	0.104	0.170	-0.230	0.437			
Age class pair Sub-adult Year 1-Juvenile : Number biweek within integration period[1]	0.178	0.166	-0.148	0.503			
Age class pair Sub-adult Year 1-Sub-adult Year 1 : Number biweek within integration period[1]	-0.102	0.164	-0.425	0.220			
Age class pair Sub-adult Year 1-Sub-adult Year 2 : Number biweek within integration period[1]	-0.063	0.171	-0.399	0.273			
Age class pair Sub-adult Year 2-Adult : Number biweek within integration period[1]	0.108	0.195	-0.274	0.490			
Age class pair Sub-adult Year 2-Juvenile : Number biweek within integration period[1]	0.153	0.174	-0.188	0.494			
Age class pair Sub-adult Year 2-Sub-adult Year 1 : Number biweek within integration period[1]	0.022	0.172	-0.315	0.359			
Age class pair Sub-adult Year 2-Sub-adult Year 2 : Number biweek within integration period[1]	-0.072	0.222	-0.508	0.364			

Age class pair Adult-Juvenile : Overlap type Overlap_95 : Number biweek within integration period[1]	-0.187	0.191	-0.560	0.187			
Age class pair Adult-Sub-adult Year 1 : Overlap type Overlap_95 : Number biweek within integration period[1]	-0.289	0.199	-0.679	0.101			
Age class pair Adult-Sub-adult Year 2 : Overlap type Overlap_95 : Number biweek within integration period[1]	-0.319	0.238	-0.785	0.147			
Age class pair Juvenile-Adult : Overlap type Overlap_95 : Number biweek within integration period[1]	-0.119	0.190	-0.491	0.254			
Age class pair Juvenile-Juvenile : Overlap type Overlap_95 : Number biweek within integration period[1]	0.091	0.180	-0.262	0.443			
Age class pair Juvenile-Sub-adult Year 1 : Overlap type Overlap_95 : Number biweek within integration period[1]	-0.138	0.182	-0.495	0.219			
Age class pair Juvenile-Sub-adult Year 2 : Overlap type Overlap_95 : Number biweek within integration period[1]	-0.176	0.196	-0.560	0.208			
Age class pair Sub-adult Year 1-Adult : Overlap type Overlap_95 : Number biweek within integration period[1]	-0.142	0.198	-0.531	0.246			
Age class pair Sub-adult Year 1-Juvenile : Overlap type Overlap_95 : Number biweek within integration period[1]	-0.035	0.182	-0.391	0.322			
Age class pair Sub-adult Year 1-Sub-adult Year 1 : Overlap type Overlap_95 : Number biweek post CBJ release[1]	-0.057	0.189	-0.426	0.313			
Age class pair Sub-adult Year 1-Sub-adult Year 2 : Overlap type Overlap_95 : Number biweek within integration period[1]	-0.111	0.205	-0.512	0.291			
Age class pair Sub-adult Year 2-Adult : Overlap type Overlap_95 : Number biweek within integration period[1]	-0.198	0.242	-0.671	0.276			
Age class pair Sub-adult Year 2-Juvenile : Overlap type Overlap_95 : Number biweek within integration period[1]	-0.038	0.196	-0.422	0.347			
Age class pair Sub-adult Year 2-Sub-adult Year 1 : Overlap type Overlap_95 : Number biweek within integration period[1]	-0.073	0.204	-0.473	0.328			
Age class pair Sub-adult Year 2-Sub-adult Year 2 : Overlap type Overlap_95 : Number biweek within integration period[1]	-0.092	0.279	-0.639	0.455			
**Family size pair Large-Small**	**-0.452**	**0.278**	**-0.997**	**0.093**	**4.847**	**3**	**0.183**
**Family size pair Small-Large**	**0.342**	**0.238**	**-0.125**	**0.808**			
**Family size pair Small-Small**	**-0.094**	**0.366**	**-0.811**	**0.623**			
**Sibling status Yes**	**0.121**	**0.069**	**-0.014**	**0.255**	**3.090**	**1**	**0.079**
**Release group type Same**	**0.246**	**0.046**	**0.157**	**0.335**	**29.060**	**1**	**<0.001*** **

Table S12: Model results for effect of interaction of release group type and sibling status with time within integration period (in days) and family size pair (QIII) on the interindividual roosting distance (Model 3b); related to Results and Figure 11. Test fixed predictors are indicated in bold. Reference categories are “female-female” for Sex pair; “Adult-Adult” for Age class pair; “Large-Large” for Family size pair; “Different” for Release group type; and “No” for Sibling status. [1]This covariate was z-transformed before including in the model.

**Model 3b: Inter-individual roosting distance (beta GLMM)**							
**Term**	**Estimate**	**SE**	**95% CL_{lower}**	**95% CL_{upper}**	**χ^2**	**df**	**p-value**
(Intercept)	-0.158	0.398	-0.938	0.623			
Sex pair female-male	0.356	0.139	0.084	0.628			
Sex pair male-male	0.545	0.255	0.046	1.044			
Age class pair Juvenile-Adult	-0.785	0.131	-1.042	-0.527			
Age class pair Juvenile-Juvenile	-2.174	0.174	-2.514	-1.833			
Age class pair Juvenile-Sub-adult Year 1	-1.628	0.150	-1.922	-1.335			
Age class pair Juvenile-Sub-adult Year 2	-1.360	0.136	-1.626	-1.094			
Age class pair Sub-adult Year 1-Adult	-1.021	0.097	-1.212	-0.830			
Age class pair Sub-adult Year 1-Sub-adult Year 1	-0.769	0.138	-1.040	-0.498			
Age class pair Sub-adult Year 1-Sub-adult Year 2	-0.681	0.116	-0.908	-0.454			
Age class pair Sub-adult Year 2-Adult	-0.634	0.074	-0.778	-0.490			
Age class pair Sub-adult Year 2-Sub-adult Year 2	-0.667	0.117	-0.896	-0.439			
Number days within integration period[1]	-0.081	0.159	-0.391	0.230			
Age class pair Juvenile-Adult : Number days within integration period[1]	-0.049	0.101	-0.246	0.149			
Age class pair Juvenile-Juvenile : Number days within integration period[1]	-0.177	0.116	-0.404	0.051			
Age class pair Juvenile-Sub-adult Year 1 : Number days within integration period[1]	0.367	0.110	0.153	0.582			
Age class pair Juvenile-Sub-adult Year 2 : Number days within integration period[1]	0.047	0.101	-0.151	0.246			
Age class pair Sub-adult Year 1-Adult : Number days within integration period[1]	0.293	0.082	0.132	0.455			
Age class pair Sub-adult Year 1-Sub-adult Year 1 : Number days within integration period[1]	0.450	0.096	0.262	0.638			
Age class pair Sub-adult Year 1-Sub-adult Year 2 : Number days within integration period[1]	0.413	0.092	0.234	0.593			
Age class pair Sub-adult Year 2-Adult : Number days within integration period[1]	0.186	0.074	0.041	0.330			

Age class pair Sub-adult Year 2-Sub-adult Year 2 : Number days within integration period[1]	-0.011	0.098	-0.203	0.181			
**Family size pair Large-Small**	**0.139**	**0.137**	**-0.130**	**0.408**	**2.733**	**2**	**0.255**
**Family size pair Small-Small**	**0.368**	**0.259**	**-0.139**	**0.875**			
**Release group type Same**	**-0.515**	**0.080**	**-0.672**	**-0.357**			
**Release group type Same : Number days within integration period[1]**	**0.166**	**0.057**	**0.054**	**0.278**	**8.449**	**1**	**<0.001*****
**Sibling status Yes**	**-0.298**	**0.135**	**-0.562**	**-0.033**			
**Sibling status Yes : Number days within integration period[1]**	**0.053**	**0.092**	**-0.128**	**0.234**	**0.331**	**1**	**0.565**

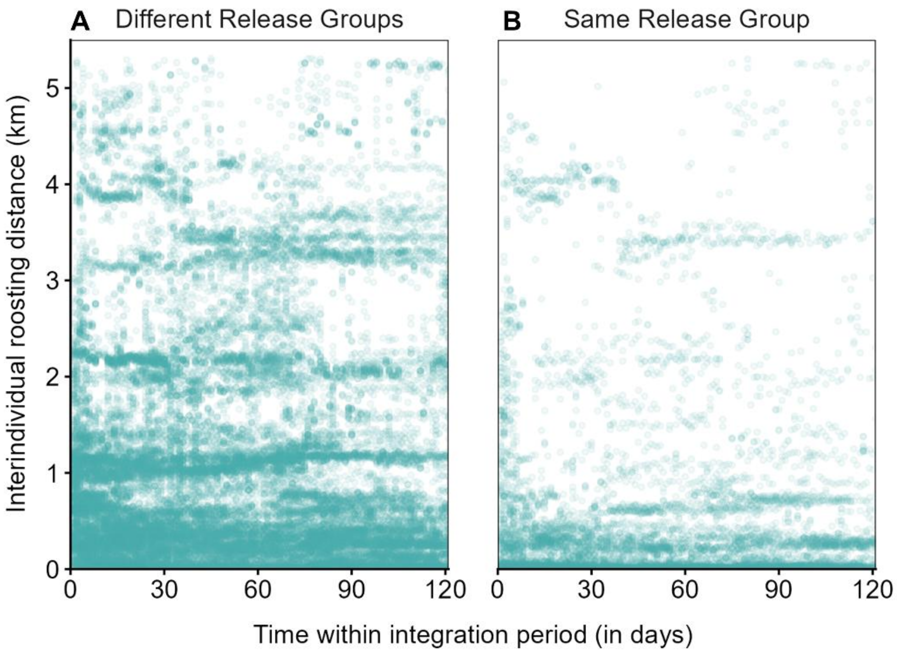

Figure S7: Observed data distribution for interindividual roosting distances (response variable 3) for dyads of individuals from different (**A**) or same (**B**) release over time within the integration period; related to Figure 11

Note S1: System and R session information, related to STAR Methods

The statistical analyses for Models 1a, 1b, 2a, 2b, 2c, and 2d, all model diagnostics, and data visualization were carried out in R (version 4.3.2) on a Windows 11 Pro, 64-bit system with an Intel i7 Processor. The package ‘glmmTMB’ (version 1.1.8) was used for fitting the GLMMs.
R Session Information for the Windows system:

```
R version 4.3.2 (2023-10-31 ucrt)
Platform: x86_64-w64-mingw32/x64 (64-bit)
Running under: Windows 11 x64 (build 26100)

Matrix products: default


locale:
[1] LC_COLLATE=English_United Kingdom.utf8
[2] LC_CTYPE=English_United Kingdom.utf8
[3] LC_MONETARY=English_United Kingdom.utf8
[4] LC_NUMERIC=C
[5] LC_TIME=English_United Kingdom.utf8

attached base packages:
[1] stats     graphics  grDevices utils     datasets  methods   base

other attached packages:
 [1] gghalves_0.1.4  DHARMa_0.4.6    lme4_1.1-35.1   Matrix_1.6-5
 [5] car_3.1-2       carData_3.0-5   dplyr_1.1.4     ggplot2_3.5.1
 [9] ggeffects_1.7.0 tictoc_1.2      glmmTMB_1.1.8
```

The statistical analyses for Models 3a and 3b were carried out on a Linux Ubuntu 22.04.5 LTS, 64-bit system using R (version 4.1.2) and ‘glmmTMB’ (version 1.1.9). R session information for the Ubuntu system:

```
R version 4.1.2 (2021-11-01)
Platform: x86_64-pc-linux-gnu (64-bit)
Running under: Ubuntu 22.04.5 LTS

Matrix products: default
BLAS:   /usr/lib/x86_64-linux-gnu/blas/libblas.so.3.10.0
LAPACK: /usr/lib/x86_64-linux-gnu/lapack/liblapack.so.3.10.0

locale:
 [1] LC_CTYPE=en_US.UTF-8       LC_NUMERIC=C
 [3] LC_TIME=de_AT.UTF-8        LC_COLLATE=en_US.UTF-8
 [5] LC_MONETARY=de_AT.UTF-8    LC_MESSAGES=en_US.UTF-8
 [7] LC_PAPER=de_AT.UTF-8       LC_NAME=C
 [9] LC_ADDRESS=C               LC_TELEPHONE=C
[11] LC_MEASUREMENT=de_AT.UTF-8 LC_IDENTIFICATION=C

attached base packages:
[1] stats     graphics  grDevices utils     datasets  methods   base

other attached packages:
[1] tictoc_1.2.1  glmmTMB_1.1.9
```

Note S2: Explanation for random slopes structure, related to STAR Methods

We initially fitted all models with the respective maximal random slopes structure (Barr et al. 2013), i.e. including all theoretically identifiable random slopes. In case of Models 1b, 2a, 2b, 2c, 2d, 3a, and 3b, the model with the maximal random slope structure failed to converge, despite increasing the number of iterations of the maximum likelihood algorithm. We thus simplified the random slope structure by excluding the random slopes that were less biologically/ecologically meaningful one at a time, until we achieved convergence. For details of the final random slope structure for the models, please refer to the R Script uploaded along with the data.

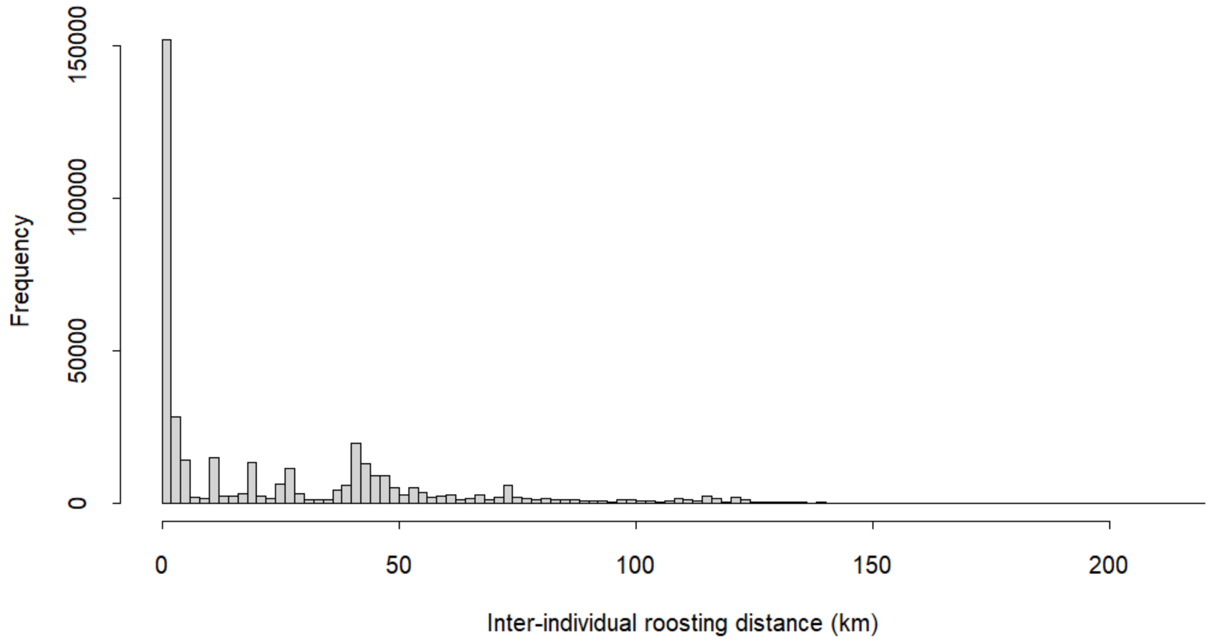

Figure S8: Histogram of the distribution of interindividual roosting distances (response variable 3), with a bin width of 2km and the right-skew clearly apparent; related to STAR Methods. Our chosen threshold of the 50% quantile (5.31km) to subset the data falls within the third bin (4-6km).

Table S13: Summary of the different response variables, choice of probability distributions, and the different diagnostic tests applied: overdispersion and variation inflation factors (VIF); related to STAR Methods. The max VIF values for all models were within the acceptable range (less than 2), indicating no collinearity issues, and the dispersion parameters suggested no overdispersion issues. Also indicated in the table are the figures corresponding to the diagnostic tests for the best linear unbiased predictors (BLUPs) for each of the models.

	Type of response	Probability distribution modelled	Dispersion parameter	max VIF	BLUPs
**Model 1a**	Discrete proportion	binomial	0.440	1.073	Figure S9
**Model 1b**			0.441	1.033	Figure S10
**Model 2a**	Binary	binomial		1.228	Figure S11
**Model 2b**				1.669	Figure S12
**Model 2c**	Continuous Proportion	beta	0.819	1.197	Figure S13
**Model 2d**			0.874	1.779	Figure S14
**Model 3a**	Lower bound, continuous, with an artificially upper bound, hence converted to a continuous proportion	beta	0.930	1.147	Figure S15
**Model 3b**			1.111	1.767	Figure S16

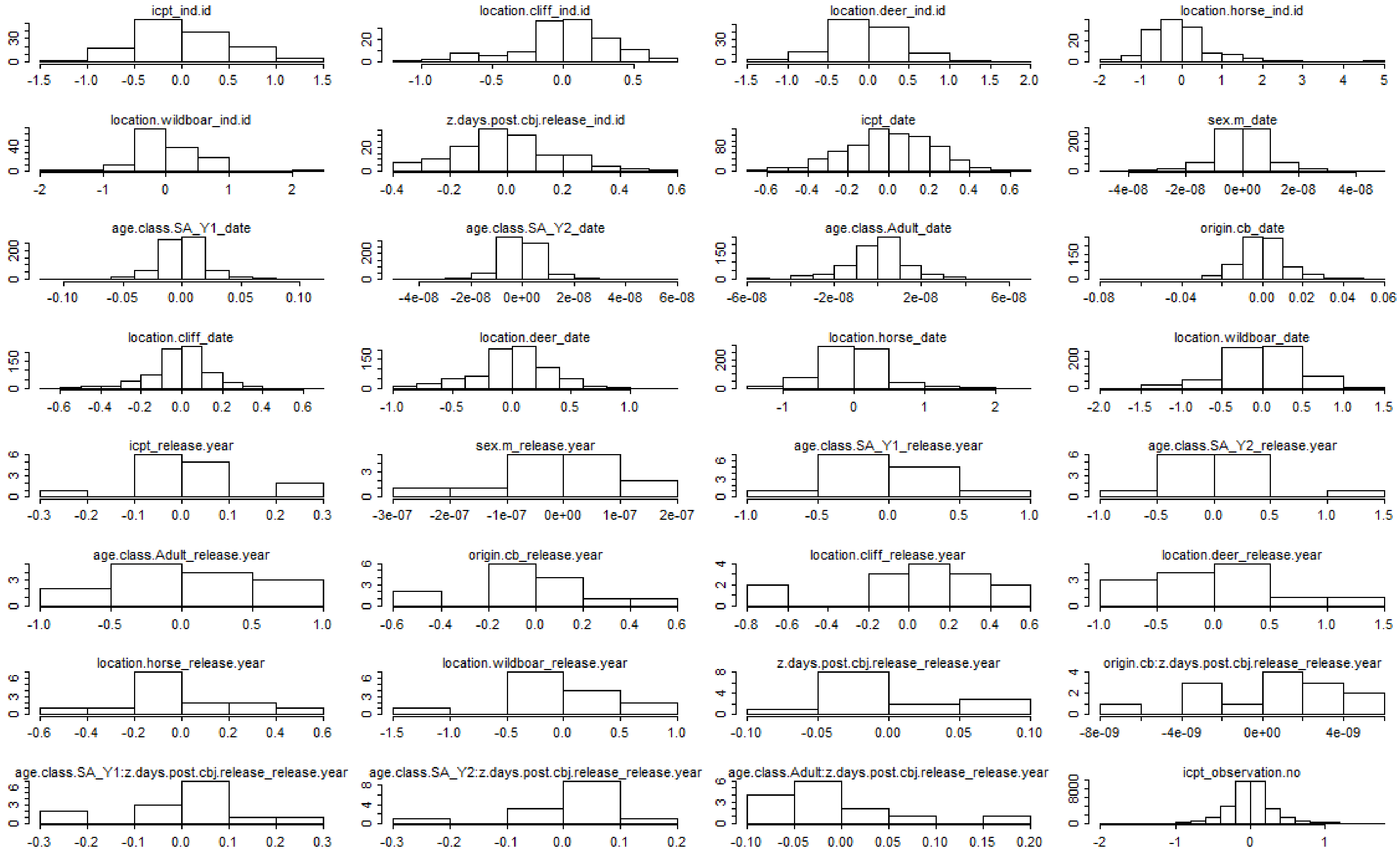

Figure S9: Distribution of best linear unbiased predictors (BLUPs) for Model 1a (probability of using common locations); related to STAR Methods. We visually inspected that the BLUPs were normally distributed with low within group variation (range of x-axis not exceeding -3 to +3) in majority of cases.

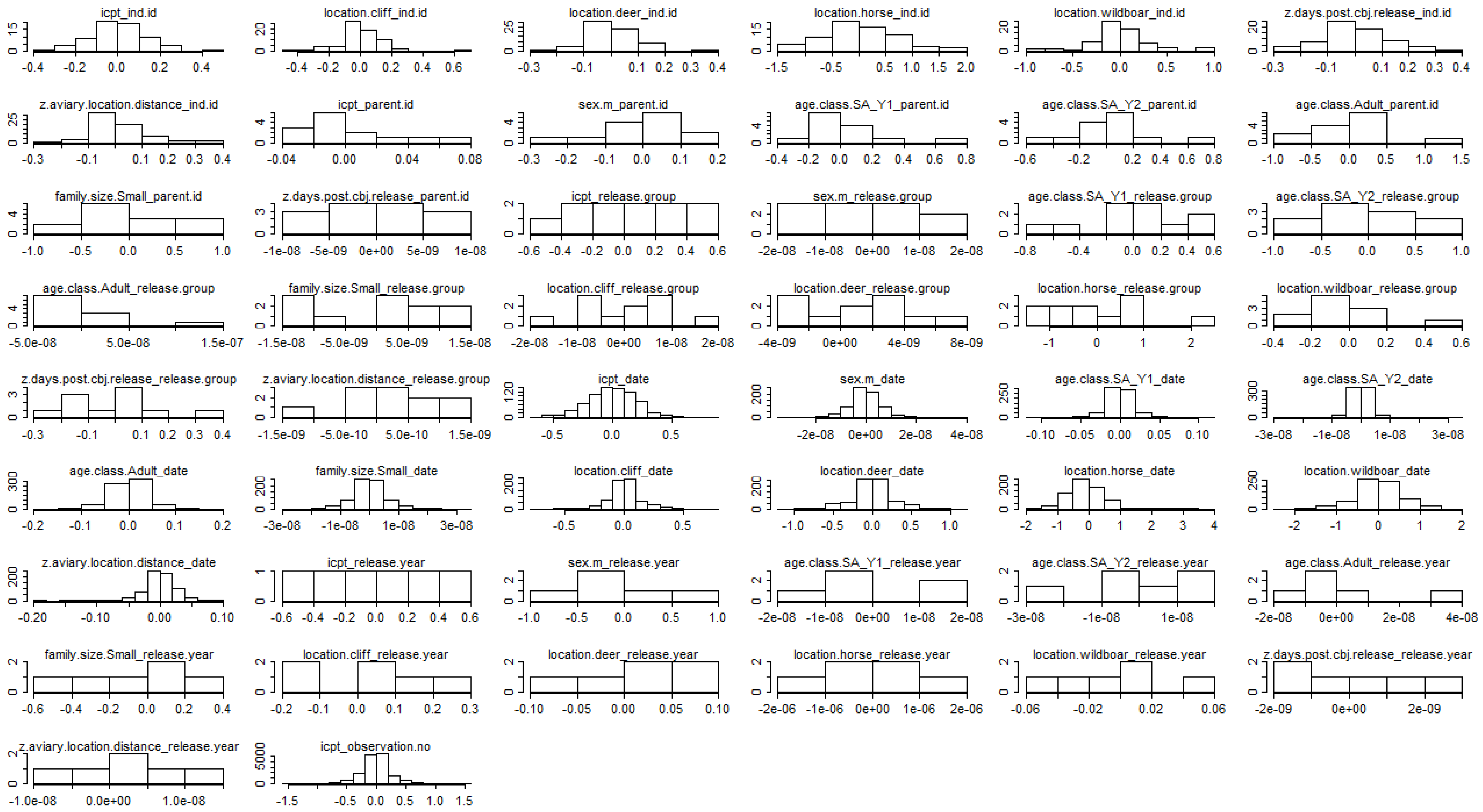

Figure S10: Distribution of best linear unbiased predictors (BLUPs) for Model 1b (probability of using common locations); related to STAR Methods. We visually inspected that the BLUPs were normally distributed with low within group variation (range of x-axis not exceeding -3 to +3) in majority of cases.

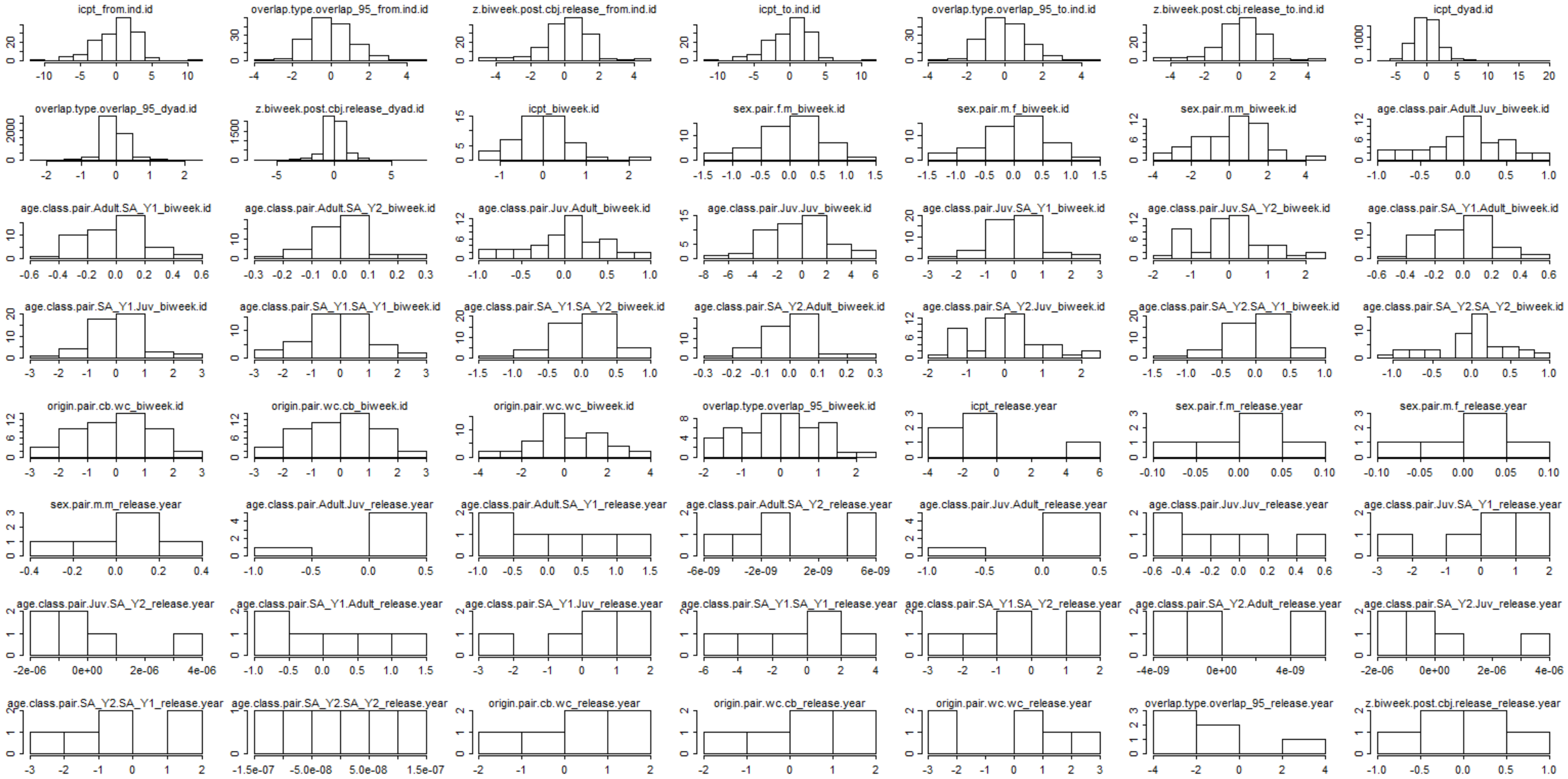

Figure S11: Distribution of best linear unbiased predictors (BLUPs) for Model 2a (occurrence of range overlap); related to STAR Methods. We visually inspected that the BLUPs were normally distributed with low within group variation (range of x-axis not exceeding -3 to +3) and found that several BLUPs show large within group variation (x-axis ranging between -5 to +20). Hence, we interpreted the model results with caution as large BLUPs can lead to unstable model estimates.

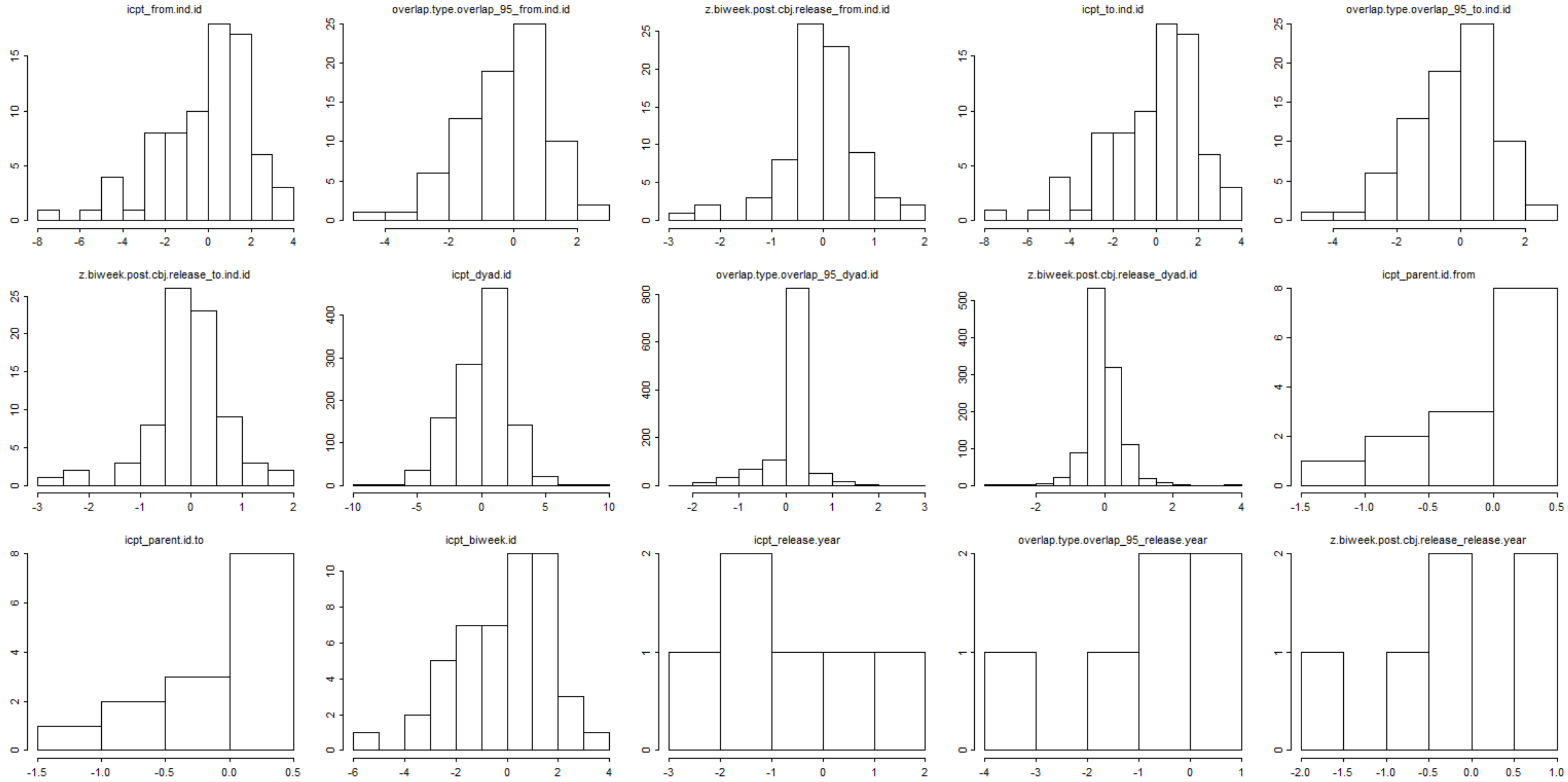

Figure S12: Distribution of best linear unbiased predictors (BLUPs) for Model 2b (occurrence of range overlap); related to STAR Methods. We visually inspected that the BLUPs were normally distributed with low within group variation (range of x-axis not exceeding -3 to +3) and found that several BLUPs show large within group variation (x-axis ranging between -10 to +10). Since this model also had cases of complete separation, we did not trust the model estimates.

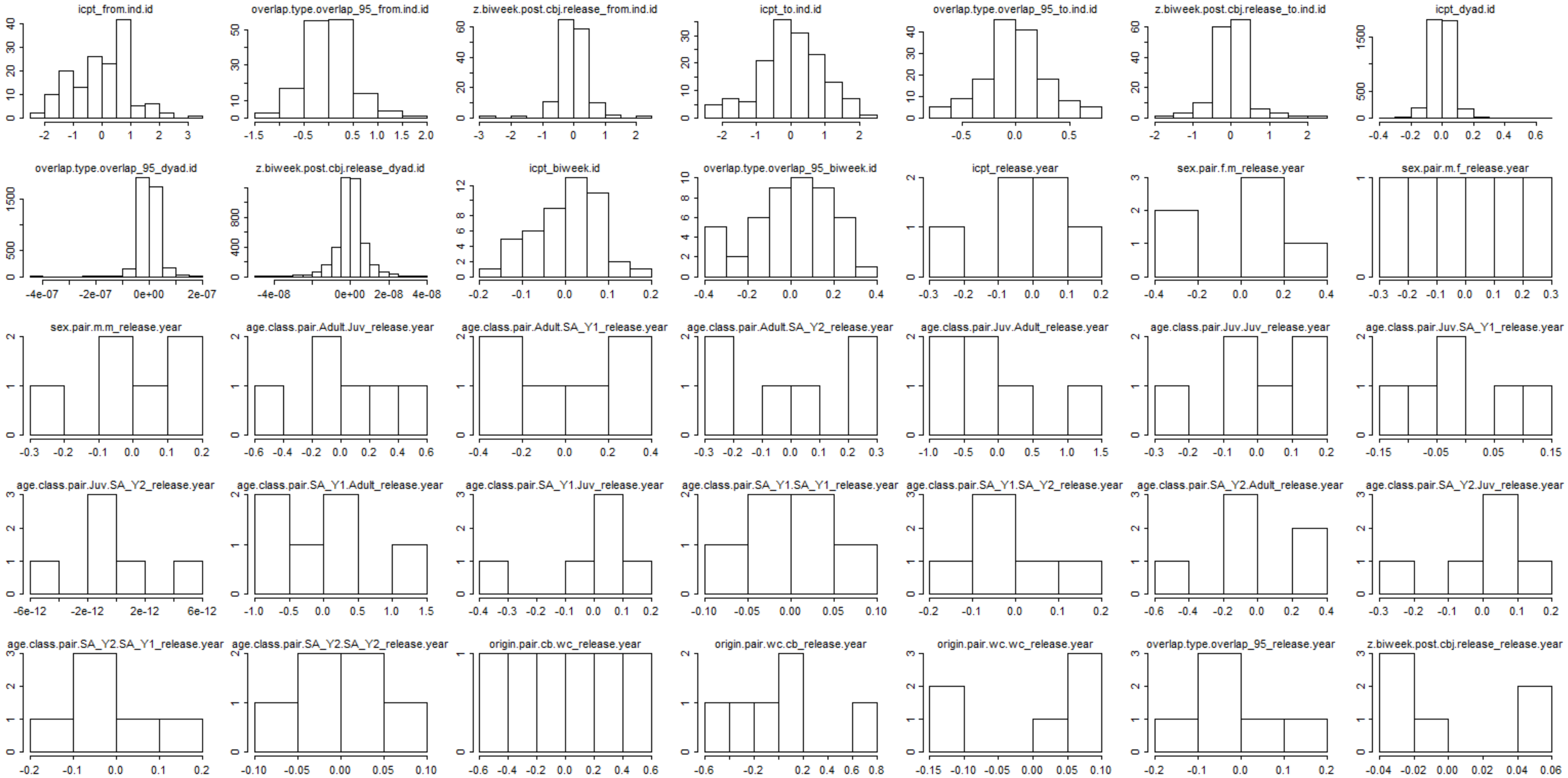

Figure S13: Distribution of best linear unbiased predictors (BLUPs) for Model 2c (range overlap); related to STAR Methods. We visually inspected that the BLUPs were normally distributed with low within group variation (range of x-axis not exceeding -3 to +3.

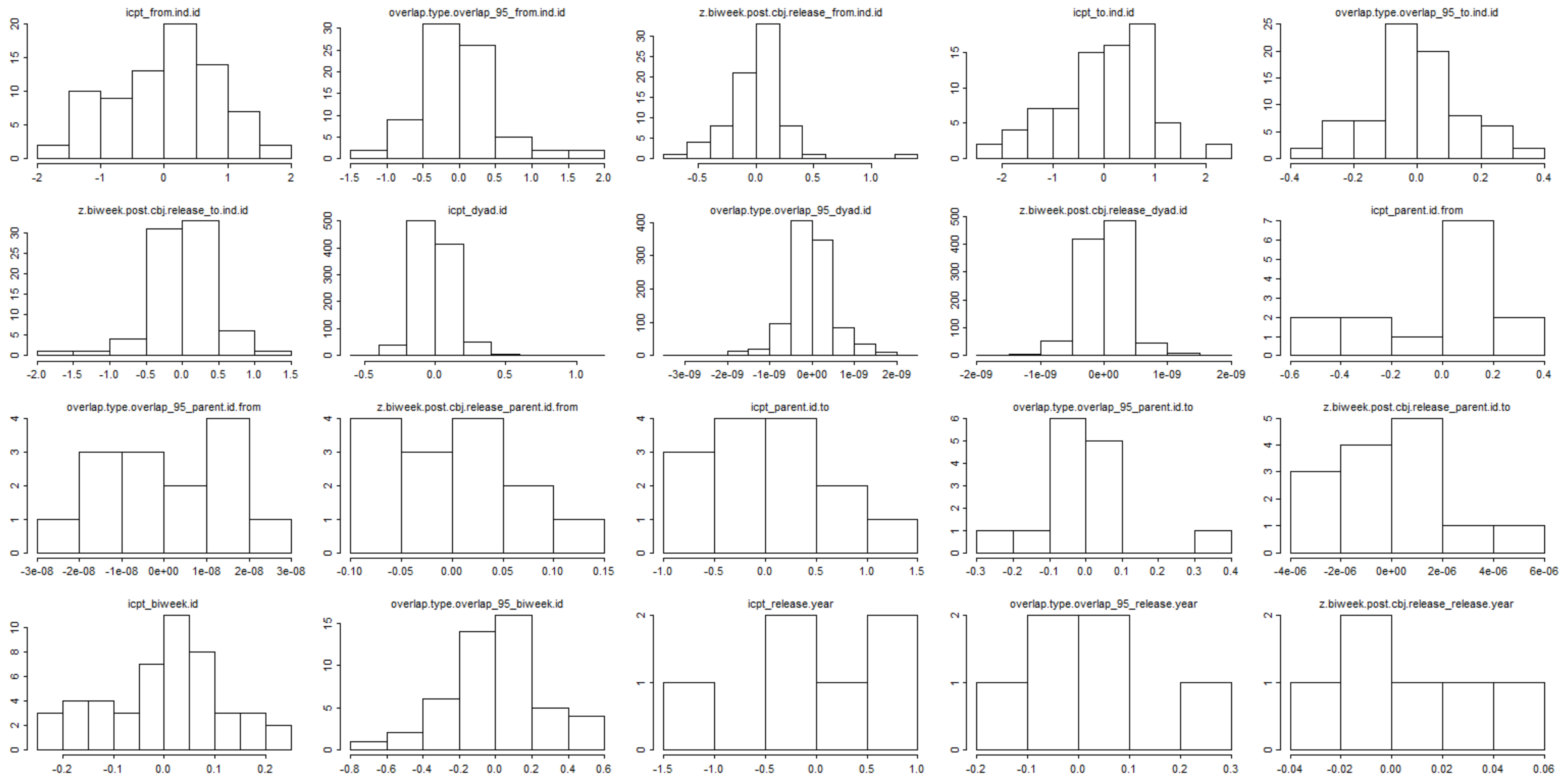

Figure S14: Distribution of best linear unbiased predictors (BLUPs) for Model 2d (range overlap); related to STAR Methods. We visually inspected that the BLUPs were normally distributed with low within group variation (range of x-axis not exceeding -3 to +3).

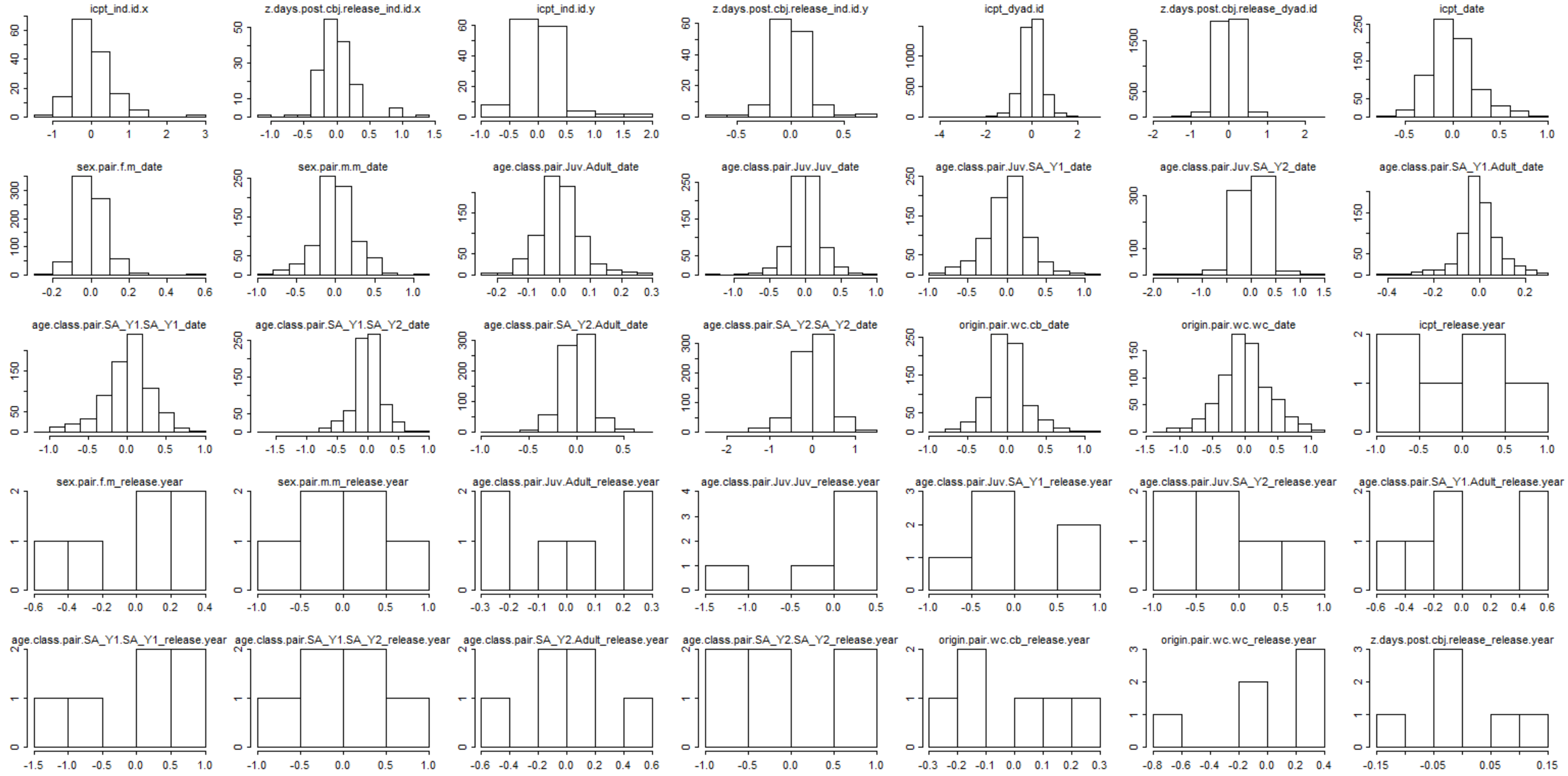

Figure S15: Distribution of best linear unbiased predictors (BLUPs) for Model 3a (interindividual roosting distance); related to STAR Methods. We visually inspected that the BLUPs were normally distributed with low within group variation (range of x-axis not exceeding -3 to +3) in majority of cases.

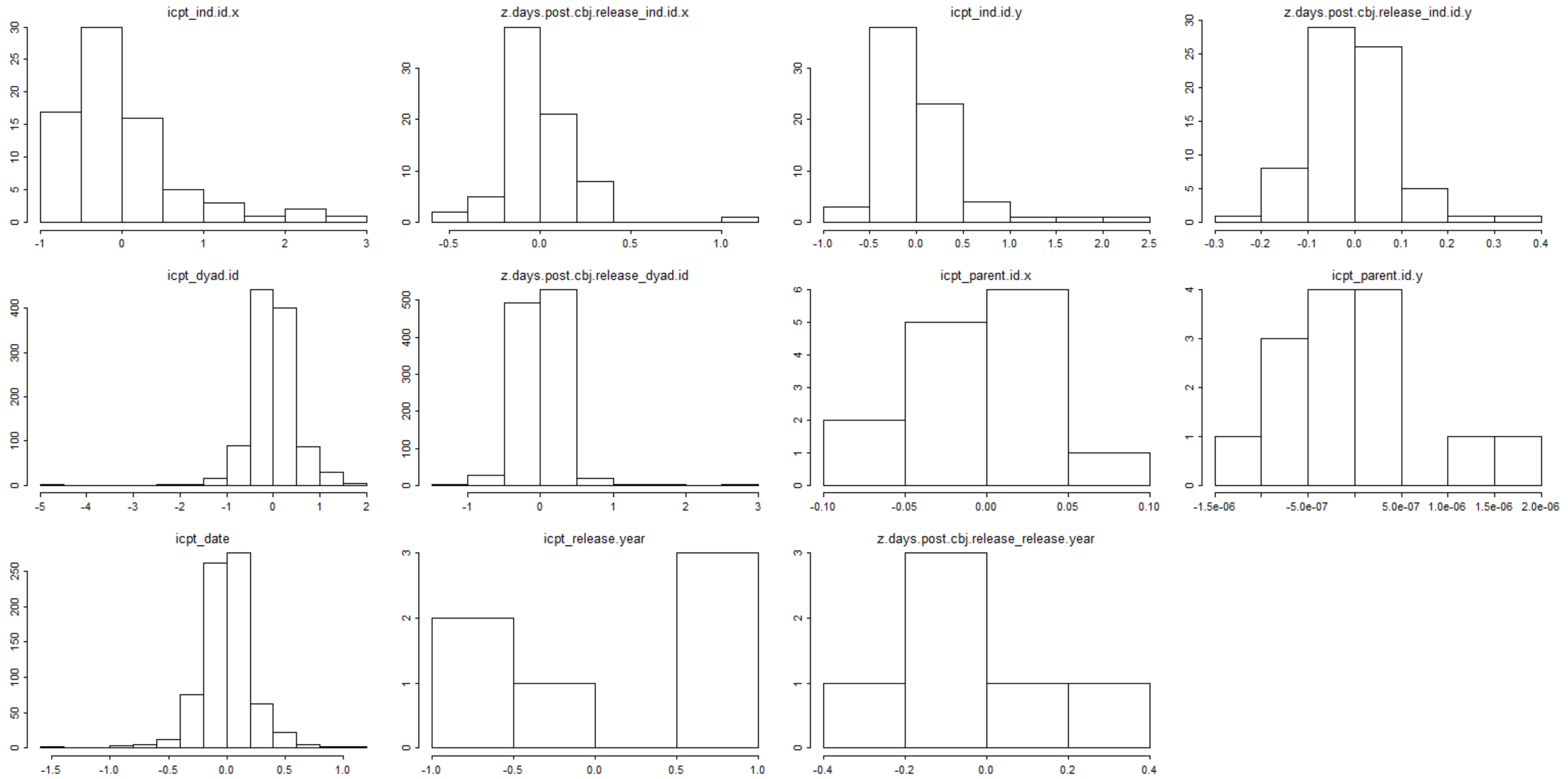

Figure S16: Distribution of best linear unbiased predictors (BLUPs) for Model 3b (interindividual roosting distance), related to STAR Methods. We visually inspected that the BLUPs were normally distributed with low within group variation (range of x-axis not exceeding -3 to +3) in majority of cases.
